# RhoB affects colitis through modulating cell signaling and intestinal microbiome

**DOI:** 10.1186/s40168-022-01347-3

**Published:** 2022-09-16

**Authors:** Jianming Yang, Geng Pei, Xuan Sun, Yawen Xiao, Chunhui Miao, Lu Zhou, Bangmao Wang, Liu Yang, Mingyu Yu, Zhi-Song Zhang, Evan T. Keller, Zhi Yao, Quan Wang

**Affiliations:** 1 Key Laboratory of Immune Microenvironment and Disease (Ministry of Education), Tianjin Institute of Immunology, Tianjin Institute of Urology, Department of Immunology, School of Basic Medical Sciences, Tianjin Medical University, 22 Qixiangtai Road, Heping District 300070, Tianjin, 300070 China; 2 Department of Gastroenterology and Hepatology, Tianjin Medical University general hospital, Tianjin Medical University, Tianjin, 300070 China; 3 State Key Laboratory of Medicinal Chemical Biology and College of Pharmacy, Collaborative Innovation Center for Biotherapy, Tianjin Key Laboratory of Molecular Drug Research, Nankai University, Tianjin, 300350 China; 4 Department of Urology, Biointerfaces Institute, University of Michigan, Ann Arbor, MI USA; 5 The Province and Ministry Co-sponsored Collaborative Innovation Center for Medical Epigenetics, Tianjin Medical University, Tianjin, 300070 China

**Keywords:** RhoB, Inflammatory bowel diseases, Microbiome, SCFAs

## Abstract

**Background:**

The pathogenesis of inflammatory bowel diseases (IBD) is multifactorial, and diagnostic and treatment strategies for IBD remain to be developed. RhoB regulates multiple cell functions; however, its role in colitis is unexplored.

**Results:**

Here, we found RhoB was dramatically increased in colon tissues of ulcerative colitis (UC) patients and mice with DSS-induced colitis. Compared with wild type mice, *RhoB*^+/−^ and *RhoB*^*−/−*^ mice developed milder DSS-induced colitis and increased goblet cell numbers and IEC proliferation. Decreased RhoB promoted goblet cell differentiation and epithelial regeneration through inhibiting Wnt signaling pathway and activating p38 MAPK signaling pathway. Moreover, increased SCFA-producing bacteria and SCFA concentrations were detected in intestinal microbiome of both *RhoB*^+/*−*^ and *RhoB*^*−/−*^ mice and upregulated SCFA receptor expression was also observed.

**Conclusions:**

Taken together, a higher level of RhoB is associated with UC, which also contributes to UC development through modulating cell signaling and altering intestinal bacterial composition and metabolites. These observations suggest that RhoB has potential as a biomarker and a treatment target for UC.

Video Abstract

**Supplementary Information:**

The online version contains supplementary material available at 10.1186/s40168-022-01347-3.

## Introduction

Inflammatory bowel diseases (IBD), including Crohn’s disease (CD) and ulcerative colitis (UC) [1], which cause chronic and debilitating inflammation in the gastrointestinal tract, are a major healthcare burden and are associated with increased incidence of colon cancer [2]. The pathogenesis of IBD is multifactorial including genetic susceptibility, intestinal barrier damage, environmental risk factors, and commensal microbiota dysbiosis [3, 4]. However, many aspects of the IBD etiology are still unclear and diagnostic and treatment strategies for IBD remain to be studied.

Epithelial injuries are the typical feature of UC [5]. Colon epithelium consists of absorptive colonocytes and specialized secretory cell types such as mucus-producing goblet cells, hormone-secreting enteroendocrine cells, and tuft cells [6]. Goblet cells are distributed between the columnar epithelium of the mucosa, which synthesize and secrete mucin Muc2 that forms a protective mucus layer [7]. The mucus layer establishes a protective physical barrier that prevents pathogens and noxious substances from reaching and damaging the epithelium [8]. Additionally, the regeneration of intestinal epithelium plays an important role in sustaining intestinal epithelial integrity and epithelial injury repair in resistance to colitis [9]. The proliferation and differentiation of the intestinal epithelium are regulated by multiple pathways including Notch, MAPK, Wnt, Hippo, and bone morphogenetic protein (BMP) signaling [10, 11].

Commensal microbiota play a crucial factor in maintaining intestinal homeostasis. The intestinal microbiota of healthy adults can inhibit the growth of opportunistic pathogens and promote a proinflammatory response against invading pathogens, while dysbiosis of intestinal microbiota can induce IBD [12, 13]. Short-chain fatty acids (SCFAs), such as acetic acid, propionic acid, butyric acid, and isobutyric acid, are major end products of intestinal microbial fermentation. SCFAs have been implicated in repairing the intestinal epithelial barrier, reducing inflammation and promoting development of immune cells [14, 15].

RhoB, a member of the small Rho GTPase family, is rapidly upregulated by stress-induced signaling events including genotoxic stress, lipopolysaccharide (LPS), inflammatory cytokines, growth factors, and toxins [16]. RhoB regulates multiple cellular processes, including vesicular transport, apoptosis, DNA repair, angiogenesis, proliferation, migration, and invasion [17, 18], and we recently reported that RhoB promoted autophagic flux to enhance clearance of uropathogenic *Escherichia coli* [19]. However, the role of RhoB in colitis remains unexplored.

In this study, using *RhoB*^+/*−*^ and *RhoB*^*−/−*^ mice, we assessed the role of RhoB in regulating intestinal homeostasis. We found that RhoB expression is increased in colons from patients with severe UC and mice with dextran sulfate sodium (DSS)-induced colitis. RhoB downregulation increases goblet cell numbers, promotes regeneration of the colonic epithelium, and changes intestinal microbiome (microbiota and metabolites), which consequently result in resistance to DSS-induced colitis. The present study indicates that RhoB has potential as a diagnostic biomarker of UC and may be a possible target for UC treatment.

## Results

### RhoB is increased in colons of patients with severe UC and mice with DSS-induced colitis

**Fig. 1:**
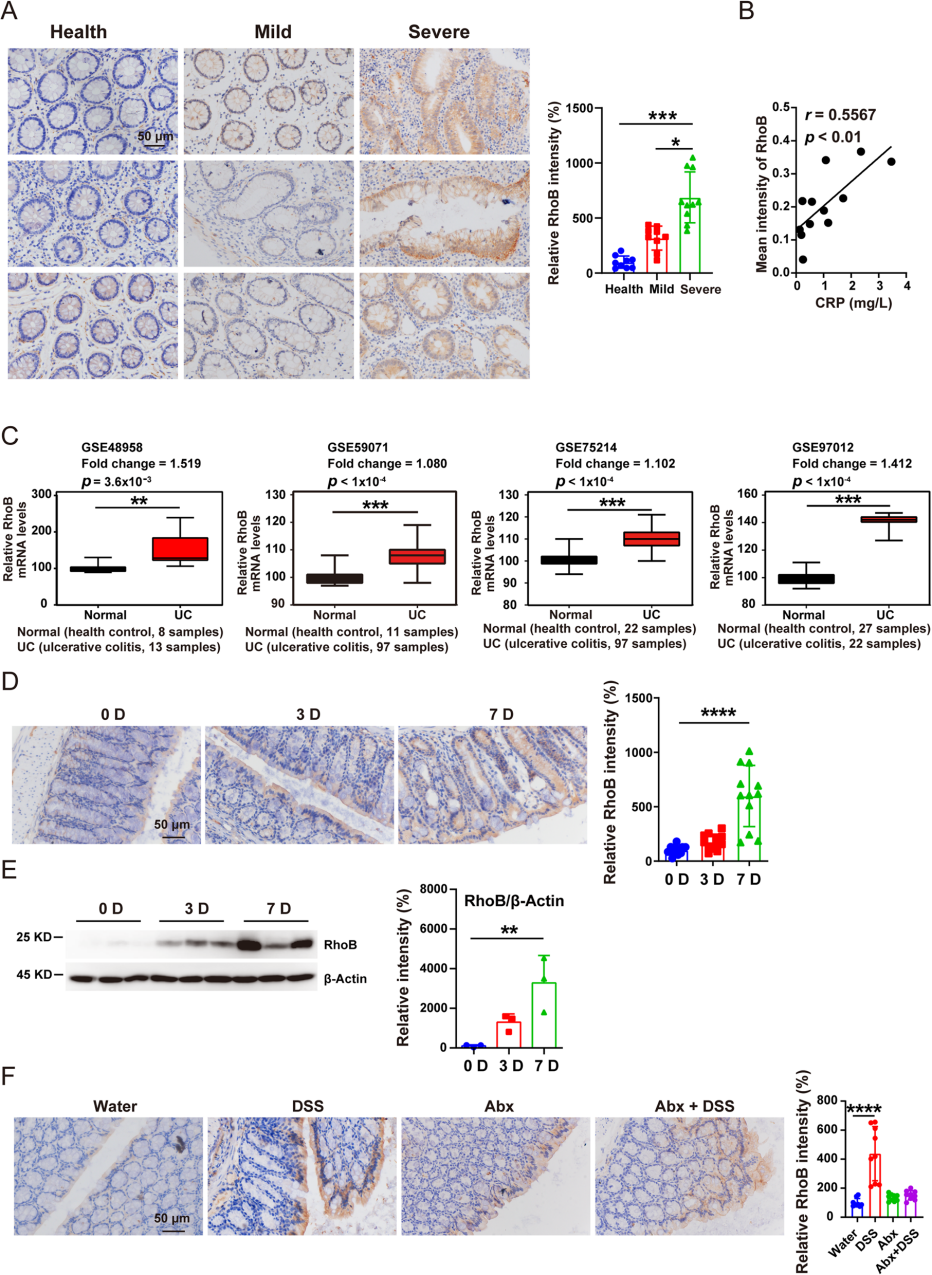
Colonic RhoB is increased in UC patients and DSS-induced colitis in mice. **A** Immunohistochemistry staining of RhoB in the colon from healthy controls (*n* = 9) and patients with mild UC (*n* = 9) and severe UC (*n* = 10). **B** Pearson’s correlation analysis of RhoB expression of intestinal mucosa and CRP (C-reactive protein) from 12 patients with UC (Spearman’s rank correlation coefficient, *r* = 0.5567. Significance is determined using linear regression, *p* < 0.01). **C** Box plot of RhoB mRNA in healthy controls and UC patients using datasets. **D** Immunohistochemistry for RhoB in the colon from C57BL/6J mice on days 0, 3, and 7 after DSS treatment. *n* = 12 from 3 independent experiments. **E** Western blotting for RhoB in the colon from WT mice on days 0, 3, and 7 after DSS treatment. *n* = 3 for each time point. **F** RhoB in colon sections from C57BL/6J mice of the water group, DSS group, Abx group, or Abx + DSS group (*n* = 9 from 3 independent experiments). Scale bar: 50 μm. Data are the mean ± SD. Statistical significance was determined by unpaired Student’s *t* test (**C**) or one-way ANOVA (**A** and **D–F**). *p < 0.05, **p < 0.01, ***p < 0.001, ****p < 0.0001

To explore the role of RhoB in UC development, we examined RhoB protein level in colonic biopsy specimens from healthy controls and patients with mild and severe UC. The immunohistochemical analysis revealed that RhoB was dramatically higher in colon tissues of severe colitis samples compared with that in healthy controls and mild colitis samples (Fig. 1A). The protein level of RhoB was positively correlated to the level of serum C-reactive protein (a widely used biomarker of inflammation in patients with UC) (Fig. 1B). To expand our observations, we also analyzed several public datasets, which revealed that RhoB mRNA was increased in UC specimens compared with that of healthy controls (NCBI’s Gene Expression Omnibus: GSE97012, GSE59071, GSE48958, and GSE75214) (Fig. 1C). These results indicate that RhoB expression is increased in the colon of UC patients.

Using available single-cell RNA sequencing (scRNAseq) data from colon biopsies of UC patients (Single Cell Portal accession SCP259) [20], we found that RhoB was highly expressed in epithelial cells compared with that in immune cells (Fig. S1A and D, Additional file 1). Moreover, RhoB was significantly increased in epithelial cells but not in immune cells in colon biopsies of UC patients compared with that of healthy controls (Fig. S1B, C, E and F, Additional file 1). We then analyzed the expression and distribution of RhoB in intestinal epithelial cell compartment, and found that RhoB was mainly increased in absorptive colonocytes, goblet cells, stem cells, and enteroendocrine cells of UC patients (Fig. S1G-I, Additional file 1). Consistent with these findings, immunofluorescent staining of colon tissues of DSS-treated mice showed that RhoB was expressed in CA1-positive colonocytes, Muc2-positive goblet cells, Lgr5-positive stem cells, and CHGA-positive enteroendocrine cells (Fig. S1J, Additional file 1).

To further define this relevance between RhoB and UC, we examined RhoB level in DSS-induced colitis in C57BL/6J mice. We found that RhoB protein level was dramatically elevated in colonic epithelia of DSS-induced colitis mice (Fig. 1D to E). To determine whether intestinal microbiota impacted RhoB expression, we treated WT mice with a cocktail of antibiotics (Abx) in drinking water before DSS challenge. Abx treatment diminished the induction of RhoB expression that was induced by DSS (Fig. 1F), implying that intestinal microbiota contribute to the increased RhoB expression by DSS treatment.

Together, these results suggest the possibility of a link between RhoB protein level in colonic epithelia and UC development.

### Decreased RhoB ameliorates DSS-induced acute and chronic colitis symptoms

**Fig. 2:**
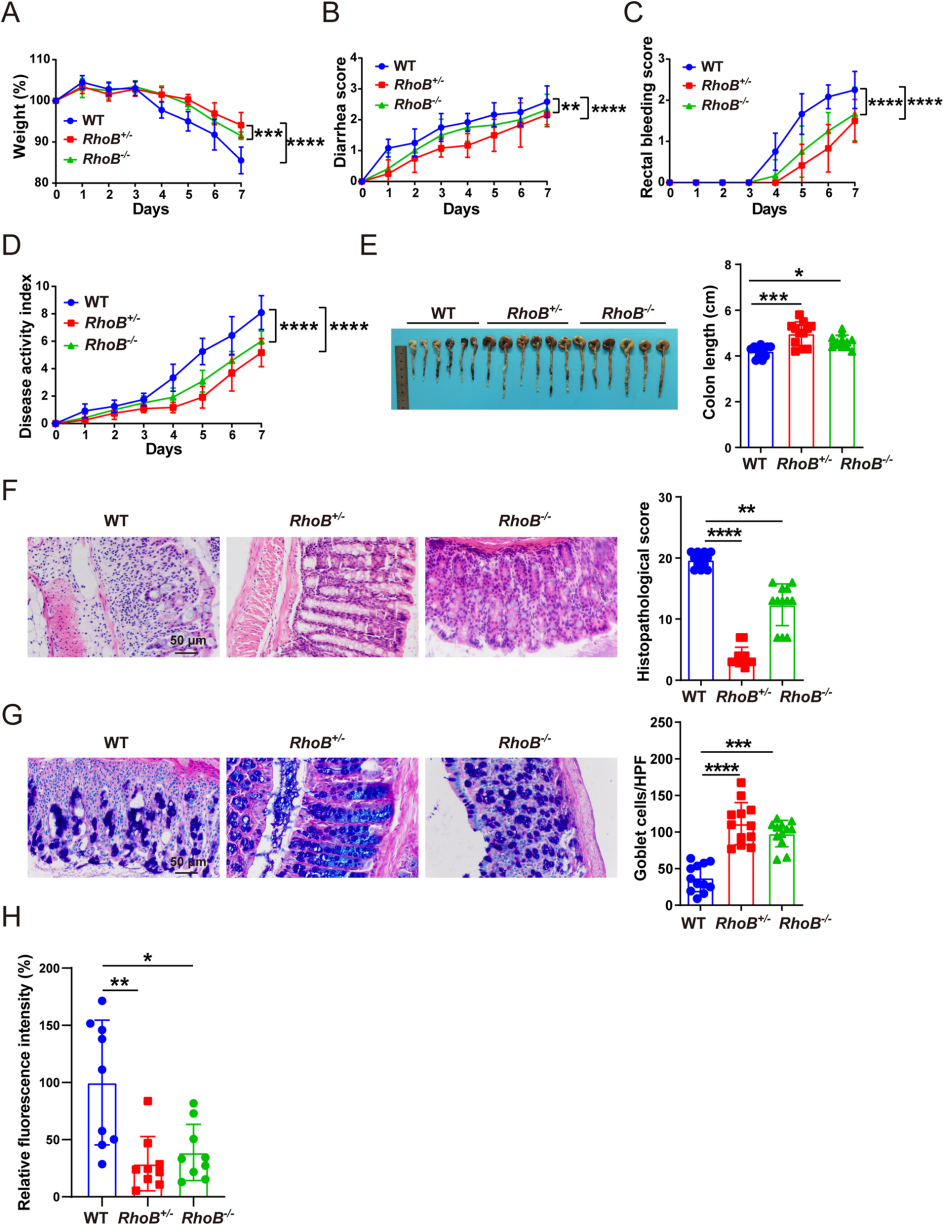
RhoB deficiency protects mice from DSS-induced colitis. **A–G** Eight-week-old male mice were given 1.5% DSS in drinking water for 7 days (*n* = 12 from 3 independent experiments). **A** Body weight loss of WT and *RhoB*^*+/−*^/*RhoB*^*−/−*^ mice after DSS treatment. **B** Diarrhea scores of WT and *RhoB*^*+/−*^/*RhoB*^*−/−*^ mice after DSS treatment. **C** Rectal bleeding scores of WT and *RhoB*^*+/−*^/*RhoB*^*−/−*^ mice after DSS treatment. **D** Disease activity index of WT and *RhoB*^*+/−*^/*RhoB*^*−/−*^ mice after DSS treatment. **E** Measurement and quantification of colon length in WT and *RhoB*^*+/−*^/*RhoB*^*−/−*^ mice after DSS treatment. **F** Representative H&E staining analysis of histopathological changes and quantitation of histology score in colon from DSS-exposed mice of the indicated genotypes. **G** Alcian blue-Periodic acid Schiff (AB-PAS; indicating goblet cells) staining in colon from DSS-exposed mice of the indicated genotypes. Scale bar: 50 μm. **H** Fluorescein isothiocyanate (FITC)-dextran measurement in serum from DSS-exposed mice of the indicated genotypes (*n* = 9 from 3 independent experiments). Data are the mean ± SD. Statistical significance was determined by two-way ANOVA (**A**–**D**) or one-way ANOVA (**E–H**). *p < 0.05, **p < 0.01, ***p < 0.001, ****p < 0.0001

We examined the role of RhoB in colitis using both heterozygous RhoB-deficient mice (*RhoB*^*+/−*^) and homozygous RhoB-deficient mice (*RhoB*^*−/−*^) (Fig. S1K and L, Additional file 1). We induced acute colitis using DSS in WT, *RhoB*^+/*−*^, and *RhoB*^*−/−*^ mice. Decreased weight loss, diarrhea, rectal bleeding, and disease activity index (DAI, a composite score used to evaluate the clinical manifestations of colitis) were observed in DSS-treated *RhoB*^*+/−*^ and *RhoB*^*−/−*^ mice compared with those in DSS-treated WT mice (Fig. 2A–D). Additionally, *RhoB*^+/*−*^ and *RhoB*^*−/−*^ mice exhibited longer colons and decreased histological score upon DSS treatment (Fig. 2E–F). Moreover, loss of goblet cells, a hallmark of UC [21], was obvious in DSS-treated WT mice compared with those in DSS-treated *RhoB*^*+/−*^ and *RhoB*^*−/−*^ mice (Fig. 2G), and lower plasma levels of FITC-conjugated dextran confirmed reduced intestinal barrier permeability in *RhoB*^+/*−*^ and *RhoB*^*−/−*^ mice (Fig. 2H).

Given the importance of intestinal epithelium in the colitis development through secretion of anti-microbial peptides and inflammatory cytokines, we measured the mRNA expression of anti-microbial peptides and proinflammatory cytokines in colon tissues after DSS treatment. The expression of anti-microbial peptides including *Reg3g* and *Reg3b* were higher in the colons of *RhoB*^*−/−*^ and *RhoB*^*+/−*^ mice than that of controls (Fig. S2A, Additional file 2). Meanwhile, lower expression of proinflammatory cytokines including *iNOS*, *Il-1β*, *Il-6*, *Tnfα*, *Ccl2,* and *Cxcl1* was observed in the colons of *RhoB*^*−/−*^ and *RhoB*^*+/−*^ mice than that of controls (Fig. S2A, Additional file 2). We further characterized the inflammatory responses in *RhoB*^*−/−*^ and *RhoB*^*+/−*^ mice. Naive *RhoB*^*−/−*^ and *RhoB*^*+/−*^ mice showed similar immune cell infiltration in mesenteric lymph nodes (MLN) and colonic lamina propria (cLP) compared with WT mice using FACS analysis (Fig. S2B and C, Additional file 2). Infiltrating lymphocytes including macrophages, dendritic cells, and neutrophils decreased a little in *RhoB*^*−/−*^ and *RhoB*^*+/−*^ mice after DSS administration with no significance (Fig. S2D, Additional file 2). Obviously, lower percentage of Th1 and Th2 cells and higher percentage of Treg cells were detected in DSS-treated *RhoB*^*−/−*^ and *RhoB*^*+/−*^ mice (Fig. S2D, Additional file 2). No significant difference for the percentage of Th17 cells was observed between the RhoB^+/*−*^/RhoB^*−*/*−*^ mice and WT mice after DSS administration (Fig. S2D, Additional file 2). These findings imply that RhoB deficiency alleviates inflammatory responses to DSS treatment.

We subsequently examined the role of RhoB in chronic colitis induced by lower doses of DSS. Considering that *RhoB*^+/*−*^ mice had better effect on alleviating colitis symptoms than *RhoB*^*−*/*−*^ mice, we investigated the role of RhoB in chronic colitis using *RhoB*^+/*−*^ mice. As expected, *RhoB*^+/*−*^ mice exhibited mild colitis in comparison with WT littermates, as reflected by body weight loss, disease activity index, colon length, histological score, and goblet cell numbers (Fig. S3A to F, Additional file 3).

Taken together, these results demonstrate that decreased RhoB has a potent preventive effect on DSS-induced acute and chronic colitis.

### RhoB^−/−^ enhances goblet cell numbers and promotes regeneration of the colonic epithelium

**Fig. 3:**
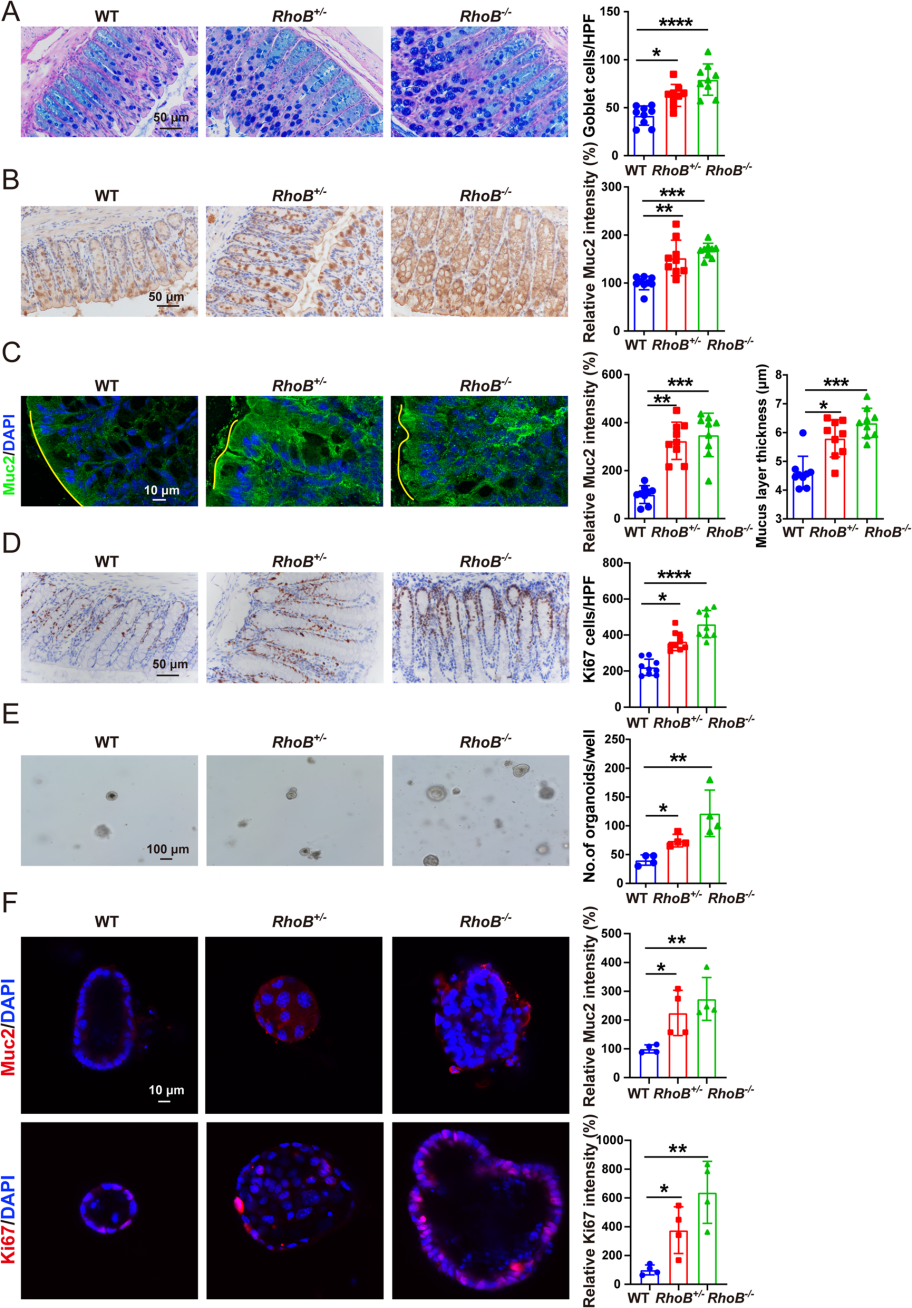
RhoB deficiency promotes intestinal homeostasis. **A** Alcian blue-Periodic acid Schiff (AB-PAS; indicating goblet cells) staining in colonic tissues of the indicated genotypes. Scale bar: 50 μm. **B** Muc2 staining in colonic tissues of the indicated genotypes. Scale bar: 50 μm. **C** Representative confocal images of Muc2 staining (green) and DAPI (blue) in colonic tissues of the indicated genotypes. Quantification of the fluorescence density and inner mucus layer thickness measured from the top of the villi to the periphery of the Muc2-positive region. Scale bar: 10 μm. **D** Ki67 staining in colon sections of the indicated genotypes. Scale bar: 50 μm. **E** Bright-field images of colonic organoids and quantitation. Colonic organoids derived from the WT or *RhoB*^+/*−*^ and *RhoB*^*−*/*−*^ mice. After 10 days of culturing, the number of organoids per well was counted. Scale bars: 100 μm. **F** Confocal images of Muc2 and Ki67 staining and quantitation in colonic organoids of the indicated genotypes. Muc2 or Ki67: red; DAPI: blue. Scale bar: 10 μm. *n* = 9 from 3 independent experiments (**A–D**), *n* = 4 from 4 independent experiments (**E–F**). Data are the mean ± SD. One-way ANOVA (**A–F**). *p < 0.05, **p < 0.01, ***p < 0.001, ****p < 0.0001

To further explore the above observed protective mechanism, we examined the effects of decreased RhoB on the colon. The colon length and colonic tissue morphology was not altered in *RhoB*^+/*−*^ and *RhoB*^*−*/*−*^ mice as compared with the control mice without DSS treatment (Fig. S3 G and H, Additional file 3). However, without any treatment *RhoB*^*−/−*^ and *RhoB*^*+/−*^ mice harbored obviously higher number of goblet cells with increased Muc2 protein level and thickness of the mucus layer, compared with the control mice (Fig. 3A–C), while no difference was observed for the levels of proteins associated with stemness (Lgr5) and tight junction (ZO-1 and Occludin) (Fig. S3I, Additional file 3). Epithelial cell proliferation and apoptosis contribute to intestinal barrier integrity. We observed that the number of Ki67^+^ cells was markedly increased in *RhoB*^*−*/*−*^ and *RhoB*^*+/−*^ colonic epithelium, suggesting that the regeneration capacity of the intestinal epithelium was substantially increased (Fig. 3D), while similar numbers of cleaved caspase 3^+^ apoptotic cells were found for *RhoB*^*−*/*−*^ and wild type mice (Fig. S3I, Additional file 3).

Next, we evaluated the functions of RhoB in epithelial cells in ex vivo conditions using colonic organoids from *RhoB*^*−/−*^, *RhoB*^*+/−*^, and wild type mice. Colonic organoids derived from *RhoB*^*−/−*^ and *RhoB*^*+/−*^ mice displayed a significant increase in numbers of organoid formation compared with that from wild type mice (Fig. 3E). Consistent with the in vivo data, the number of Ki67^+^ cells and the Muc2 level were significantly increased in *RhoB*^*−/−*^ and *RhoB*^*+/−*^ organoids relative to the controls (Fig. 3F). *RhoB* knockdown using siRNA in human colorectal cancer cell line SW480 was carried out to further validate the effects of RhoB in vitro, and increased Muc2 and Ki67 protein levels in SW480 cells with *RhoB* knockdown were observed (Fig. S4A to C, Additional file 4). Collectively, these data indicate that decreased RhoB in epithelial cells enhances goblet cell numbers and promotes epithelial regeneration, which may lead to resistance to DSS-induced colitis and enhancing intestinal barrier integrity.

### RhoB exhibited effects on goblet cell differentiation and epithelial regeneration through Wnt and p38 MAPK signaling pathway

**Fig. 4:**
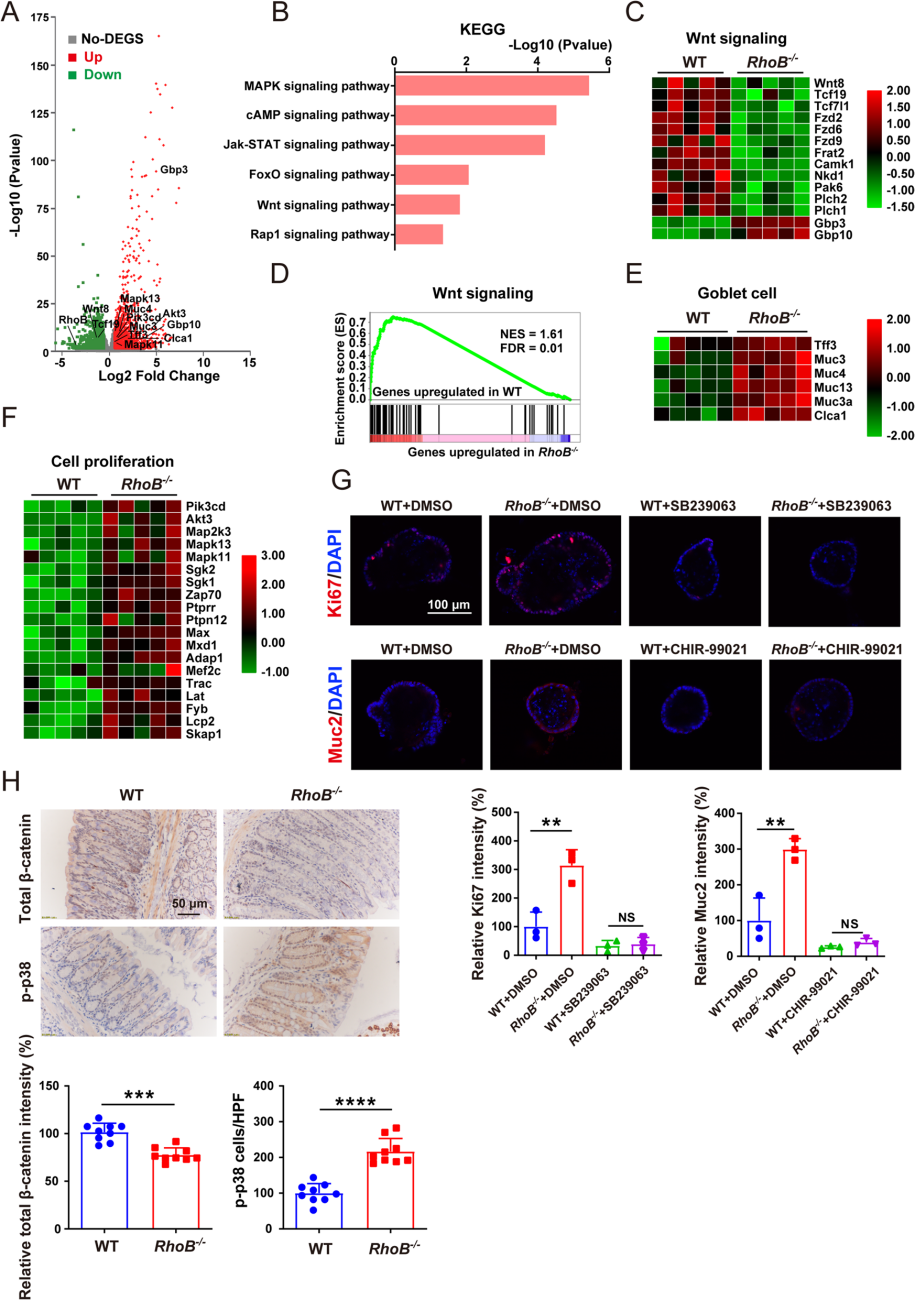
RhoB deficiency regulates Wnt and p38 MAPK signaling pathway. **A–F** RNAseq analysis of IECs from WT and *RhoB*^*−/−*^ mice. *n* = 5 for each genotype. **A** Volcano plot showing the upregulated and downregulated genes in IECs from *RhoB*^*−/−*^ mice compared with WT mice. Green indicates downregulated genes; red indicates upregulated genes; gray indicates non-differentially expressed genes (No-DEGS). **B** KEGG pathway analysis showing distinct differences in pathways between WT and *RhoB*^*−/−*^ mice. **C** Heatmap of Wnt signaling pathway-associated genes. **D** GSEA analysis of Wnt signaling pathway-associated genes in WT and *RhoB*^*−/−*^ mice. Normalized enrichment score (NES) and false discovery rates (FDR) are indicated. **E** Heatmap of goblet cell signature genes. **F** Heatmap of epithelial cells proliferation-associated genes. **G** Confocal images of Muc2 and Ki67 staining in colonic organoids of the indicated genotypes. Muc2 or Ki67: red; DAPI: blue (*n* = 3 from 3 independent experiments). Scale bar: 100 μm. **H** β-catenin and p-p38 staining in colonic tissues of the indicated genotypes (*n* = 9 from 3 independent experiments). Scale bar: 50 μm. Data are the mean ± SD. One-way ANOVA (**G**) or unpaired Student’s *t* test (**H**). **p < 0.01, ***p < 0.001, ****p < 0.0001

To elucidate the molecular mechanisms through which RhoB regulates goblet cell numbers and epithelial regeneration, we analyzed gene expression profiles of IECs from WT and *RhoB*^*−/−*^ mice. RNA sequencing analysis revealed that 1914 genes were upregulated and 1646 genes were downregulated in *RhoB*^*−/−*^ mice (*p* < 0.05 and fold change > 1.5) (Fig. 4A), which include goblet cell-related genes, goblet cell differentiation related genes, and cell proliferation-related genes (Fig. 4A, marked genes). KEGG pathway analysis showed that the differentially expressed (upregulated or downregulated) genes were mainly enriched in MAPK, Jak-STAT, cAMP, FoxO, Wnt, and Rap1 signaling pathways in *RhoB*^*−/−*^ mice (Fig. 4B). Of them, Wnt signaling pathway genes were suppressed in *RhoB*^*−/−*^ mice (Fig. 4C–D), which contributes to intestinal goblet cell differentiation. In addition, the expression levels of goblet cell signature including Tff3, Muc3, Muc4, and Clca1 were upregulated in *RhoB*^*−/−*^ mice (Fig. 4E). Simultaneously, MAPK, Jak-STAT, cAMP, FoxO, and Rap1 signaling pathways were activated in *RhoB*^*−*/*−*^ mice, which are associated with increased epithelial cell proliferation. By analyzing the individual gene expression included in these pathways, we found that the differentially expressed genes were significantly enriched in p38 MAPK and PI3K-Akt signaling pathways (Fig. 4F).

To further confirm the role of Wnt, p38 MAPK, and PI3K-Akt signaling pathway in RhoB-mediated goblet cell differentiation and epithelial cell proliferation, we treated si*RhoB* transfected SW480 cells with p38 MAPK inhibitor SB239063, PI3K/AKT inhibitor LY294002, or Wnt signaling pathway activator CHIR99021. Treatment with CHIR99021 or SB239063 rescued increased expression of Muc2 or Ki67, respectively, in si*RhoB* transfected cells (Fig. S4D to E, Additional file 4). We next treated intestinal organoids with CHIR99021 or SB239063. Muc2 and Ki67 expressions were downregulated by CHIR99021 and SB239063 in *RhoB*^*−*/*−*^ colonic organoids (Fig. 4G). We subsequently examined the expression levels of proteins associated with the Wnt and p38 MAPK signaling pathways in colon sections from *RhoB*^*−/−*^ and WT mice. Immunohistochemical analysis demonstrated lower nuclear β-catenin levels and higher phospho-p38 levels in colon sections of *RhoB*^*−/−*^ mice compared with that of WT mice (Fig. 4H). Taken together, both in vivo and in vitro findings indicate that decreased RhoB promotes goblet cell differentiation and epithelial cell proliferation through repressing the Wnt signaling pathway and activating the p38 MAPK signaling pathway.

### RhoB expression changes intestinal microbiota and promotes SCFA production

**Fig. 5:**
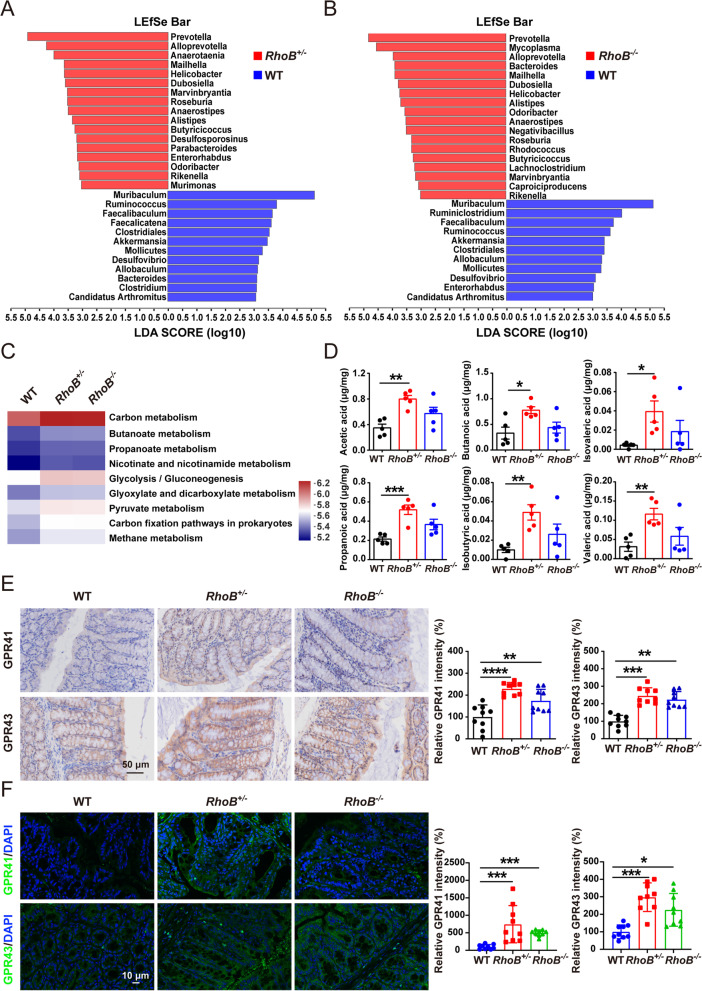
RhoB deficiency increases SCFA-producing bacteria. **A–C** Stool samples from 6–8-week-old WT and RhoB-deficient mice were collected and analyzed by 16S rRNA gene sequencing (*n* = 5). **A** LEfSe analysis of distinctive microbiota composition between WT and *RhoB*^*+/−*^ mice. **B** LEfSe analysis of distinctive microbiota composition between WT and *RhoB*^*−/−*^ mice. **C** Heatmap of KEGG pathways related with SCFAs in microbiota from WT and RhoB-deficient mice. **D** Stool samples from 6 to 8-week-old WT and RhoB-deficient mice were collected and SCFA quantification were measured by GC-MS (*n* = 5). **E** Immunohistochemical staining of GPR41 and GPR43 in colon sections as indicated (*n* = 9 from 3 independent experiments). Scale bar: 50 μm. **F** Confocal images of GPR41 and GPR43 staining in colon sections as indicated (*n* = 9 from 3 independent experiments). GPR41 or GPR43: Green; DAPI: blue. Scale bar: 10 μm. Data are the mean ± SD. One-way ANOVA (**D–F**). *p < 0.05, **p < 0.01, ***p < 0.001, ****p < 0.0001

We investigated the intestinal microbial composition of *RhoB*^*+/−*^, *RhoB*^*−/−*^, and WT mice. There was no significant difference in the amounts of bacterial DNA (Fig. S5A, Additional file 5). Analysis of the Shannon diversity index also showed no significant difference in *RhoB*^*+/−*^, *RhoB*^*−/−*^, and WT mice (Fig. S5B, Additional file 5). However, principle component analysis (PCoA) revealed major differences in the microbial composition between *RhoB*^*+/−*^/*RhoB*^*−/−*^ and WT mice (Fig. S5C, Additional file 5). We found that fecal microbiota of *RhoB*^*+/−*^/*RhoB*^*−/−*^ mice had a higher relative abundance of *Prevotella* and *Alloprevotella* and contained a lower abundance of *Muribaculum*, *Ruminococcus* and *Faecalibaculum* at the genus level (Fig. 5A, B, Fig. S5D and E, Additional file 5). These observations indicate that decreased RhoB results in intestinal microbiota alteration.

Several studies have demonstrated that *Prevotella* and *Alloprevotella* produce SCFAs, which are important metabolites in maintaining intestinal homeostasis [22, 23]. Performing KEGG pathways analysis, using PICRUSt, we found that multiple pathways involved in the metabolism of fatty acids were upregulated in *RhoB*^*+/−*^/*RhoB*^*−/−*^ mice compared with WT mice (Fig. 5C). Measurement of SCFA concentrations in fecal samples revealed a significant increase of six prominent SCFAs (acetic acid, propanoic acid, butanoic acid, isovaleric acid, isobutyric acid, and valeric acid) in *RhoB*^+/*−*^ mice compared with WT mice, and a trend, without significant difference, was also observed for *RhoB*^*−*/*−*^ mice (Fig. 5D). Furthermore, the level of SCFA concentrations were positively correlated to the relative abundance of *Prevotella* and *Alloprevotella* in fecal samples of *RhoB*^*−*/*−*^, *RhoB*^+/*−*^, and WT mice (Fig. S6A and B, Additional file 6). Immunohistochemistry and immunofluorescence analysis revealed that protein levels of SCFA receptors GPR41 and GPR43 were significantly increased in *RhoB*^*−*/*−*^ and *RhoB*^+/*−*^ mice (Fig. 5E–F). Taken together, these results demonstrate that reduced level of RhoB increases intestinal SCFA-producing bacteria and SCFA concentrations, which are beneficial microbiome for protecting against DSS-colitis.

### Intestinal microbiome are partially responsible for decreased colitis in RhoB^−/−^ and RhoB^+/−^ mice

**Fig. 6:**
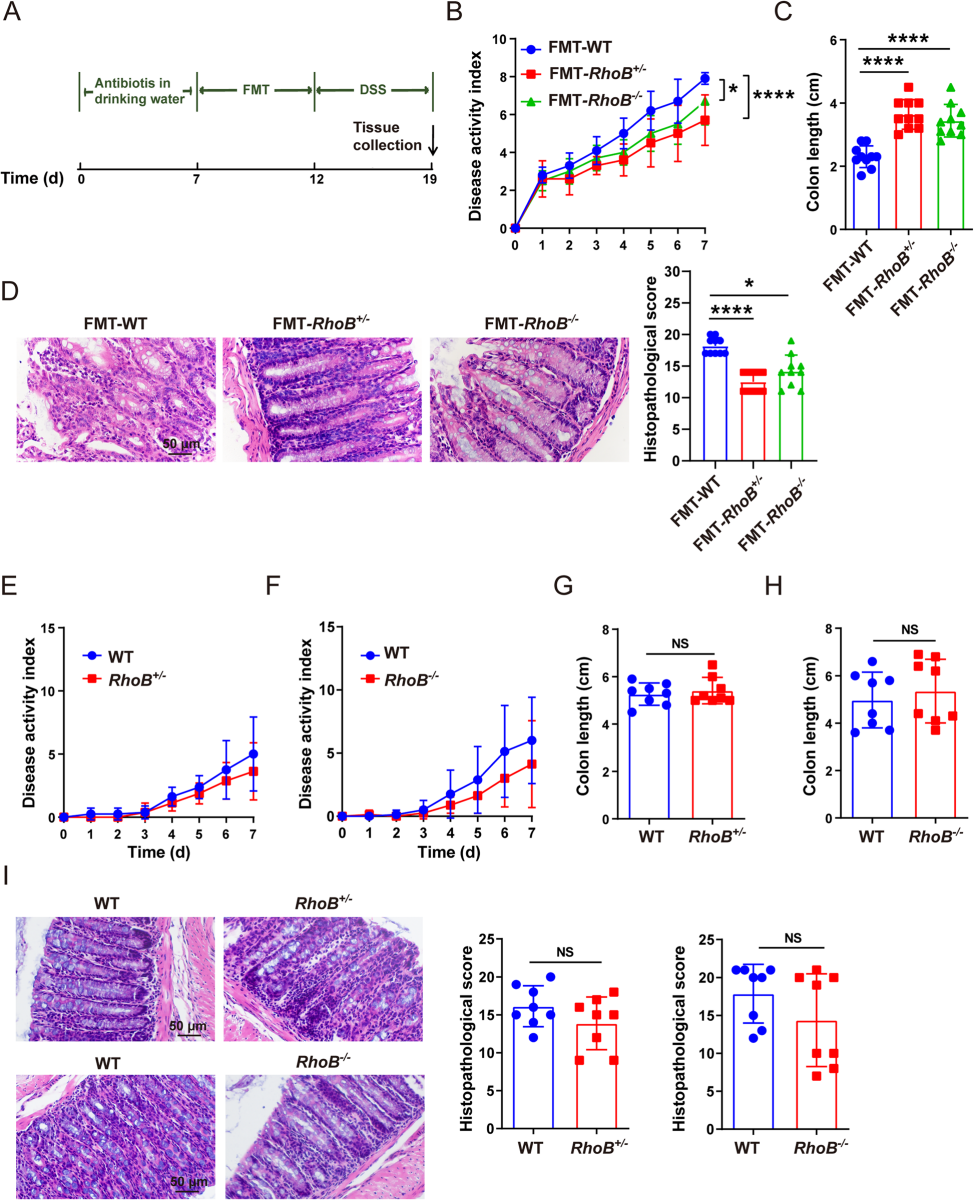
Intestinal microbiota contributes to decreased colitis in RhoB-deficient mice. **A** Schematic image illustrating FMT experimental design. **B** Disease activity index of the indicated mice and treatments. **C** Measurement and quantification of colon lengths in the indicated mice and treatments**. D** Histopathological changes and quantitation of histology score in colon of the indicated mice and treatments. *n* = 10 from 2 independent experiments (**B–D**)**. E–I** Mice were cohoused for 4 weeks and then treated with 1.5% DSS (*n* = 8 from 2 independent experiments). **E** Disease activity index of WT and *RhoB*^*+/−*^ mice. **F** Disease activity index of WT and *RhoB*^*−/−*^ mice. **G** Measurement and quantification of colon lengths in WT and *RhoB*^*+/−*^ mice. **H** Measurement and quantification of colon lengths in WT and *RhoB*^*−/−*^ mice. **I** Histopathological changes and quantitation of histology score in colon of WT, *RhoB*^*+/−*^, and *RhoB*^*+/−*^ mice. Scale bar: 50 μm. Data are the mean ± SD. Two-way ANOVA (**B**, **E**, and **F**), unpaired Student’s *t* test (**G**–**I**), or one-way ANOVA (**C**, **D**). NS, not significant. *p < 0.05, ****p < 0.0001

To determine the role of microbiome in decreased colitis, *RhoB*^*+/−*^ mice were administered a cocktail of antibiotics (Abx) in drinking water before DSS treatment (Fig. S6C, Additional file 6). Antibiotic treatment eliminated the difference in the severity of colitis including disease activity index, colon length, and histological score between WT and *RhoB*^*+/−*^ mice (Fig. S6D to F, Additional file 6). Meanwhile, we performed the fecal microbiota transplantation from *RhoB*^*+/−*^, *RhoB*^*−/−*^, and WT mice into antibiotic-treated mice (Fig. 6A). As reflected by the disease activity index, colon length and histological score, *RhoB*^*+/−*^/*RhoB*^*−/−*^ microbiota recipient mice exhibited increased resistance to colitis (Fig. 6B–D), suggesting gut microbiota contribute to the attenuated phenotype of *RhoB*^*+/−*^ and *RhoB*^*−/−*^ mice during colitis.

To further confirm our hypothesis, we cohoused WT with *RhoB*^*+/−*^ or *RhoB*^*−/−*^ mice for 4 weeks prior to DSS challenge. WT mice cohoused with *RhoB*^*+/−*^ or *RhoB*^*−/−*^ mice developed mild colitis, the similar phenotype to that of *RhoB*^*+/−*^ or *RhoB*^*−/−*^ mice, indicating a transfer of *RhoB*^*+/−*^ or *RhoB*^*−/−*^ phenotype to WT mice after cohousing (Fig. 6E–I). However, compared with WT mice, *RhoB*^*+/−*^ or *RhoB*^*−/−*^ mice after cohousing continued to show a persistent mild degree of colitis, although without significant difference (Fig. 6E–I). We further analyzed the intestinal microbial composition of *RhoB*^*+/−*^/*RhoB*^*−/−*^ and WT mice before and after cohousing. The Shannon diversity index showed no significant difference in *RhoB*^*+/−*^/*RhoB*^*−/−*^ and WT mice before and after cohousing (Fig. S7A, Additional file 7). PCoA between *RhoB*^*+/−*^/*RhoB*^*−/−*^ and WT mice were different before cohousing, while similarities were observed between *RhoB*^*+/−*^/*RhoB*^*−/−*^ and WT mice after cohousing (Fig. S7B, Additional file 7). In line with the PCoA results, the microbiota composition in the feces of WT mice shifted toward that of *RhoB*^*+/−*^/*RhoB*^*−/−*^ mice after cohousing (Fig. S7C and D, Additional file 7). Moreover, the relative abundance of *Prevotella* and *Alloprevotella* were significantly increased in microbiota of WT mice after cohousing (Fig. S7C to D, Additional file 7).

Additionally, GPR41 and GPR43 protein level were comparable between cohoused WT and *RhoB*^*−/−*^mice (Fig. S8A, Additional file 8). However, the difference in goblet cell numbers, Muc2 and Ki67 protein levels between WT and *RhoB*^*−/−*^ mice were not altered after cohousing (Fig. S8B to C, Additional file 8). These data collectively suggest that, in *RhoB*^*+/−*^ and *RhoB*^*−/−*^ mice, alteration of intestinal microbiome may partially account for the colitis phenotype, while increased goblet cells and regeneration capacity of the intestinal epithelium are not associated with altered intestinal microbiome.

### The effect of RhoB on intestinal phenotype mainly depends on its expression in epithelia

To investigate whether RhoB expression in epithelia determines its effect, we performed bone marrow transplantation. Bone marrow of CD45.1 WT was transferred into irradiated CD45.2 WT, *RhoB*^*+/−*^ or *RhoB*^*−/−*^ mice, respectively (Fig. S9A, Additional file 9), and bone marrow reconstitution was verified by analysis of CD45.1 in blood cells (Fig. S9B, Additional file 9). The colonic tissue morphology was similar between RhoB-deficient and WT recipient mice (Fig. S9C, Additional file 9). Concurring to observations in the conventional *RhoB*^*+/−*^ and *RhoB*^*−/−*^ mice (Fig. 3), *RhoB*^*+/−*^ and *RhoB*^*−/−*^ mice after bone marrow transplantation still have increased goblet cell numbers, Muc2 and Ki67 protein levels as compared with the WT recipient mice (Fig. S9D to F, Additional file 9). Moreover, GPR43 protein level was significantly increased in *RhoB*^*+/−*^ and *RhoB*^*−/−*^ recipient mice relative to WT recipient mice (Fig. S9G, Additional file 9). Overall, these data suggest that RhoB-expressing epithelia are accountable for its effect on intestinal phenotype.

### Microbiome alteration elicited by RhoB is independent of RhoB-mediated regulation of autophagic flux

We further studied how RhoB expression altered intestinal microbiome. As we found that RhoB promoted autophagic flux [19], and it is also reported that autophagy leads to alteration of the microbiota composition [24, 25], we examined whether alteration of the microbiome resulted from RhoB-mediated autophagy. WT and *RhoB*^*−/−*^ mice were treated with rapamycin (widely used to induce autophagy in mice [26]) before DSS challenge. Rapamycin treatment was found to aggravate the severity of colitis in both WT and *RhoB*^*−/−*^ mice (Fig. S8D to F, Additional file 8). However, rapamycin treatment did not abolish the difference in the severity of colitis between WT and *RhoB*^*−/−*^ mice (Fig. S8D to F, Additional file 8), suggesting that the colitis phenotype in *RhoB*^*−/−*^ mice is independent of autophagy. We further analyzed the intestinal microbiota of WT and *RhoB*^*−*/*−*^ mice after rapamycin treatment. The Shannon diversity index did not differ between WT and *RhoB*^*−/−*^ mice after rapamycin treatment (Fig. S10A, Additional file 10). PCoA analysis exhibited a clear difference between *RhoB*^*−/−*^ and WT mice after rapamycin treatment (Fig. S10B, Additional file 10). We found that the relative abundance of *Prevotella* was significantly decreased in RhoB^*−*/*−*^ mice after rapamycin treatment (Fig. S10C to E, Additional file 10). However, *RhoB*^*−*/*−*^ mice with rapamycin treatment still exhibited a higher relative abundance of *Alloprevotella*, compared with WT mice with rapamycin treatment (Fig. S10C to E, Additional file 10). In addition, goblet cells, Muc2 protein level, and thickness of the inner mucus layer in *RhoB*^*−*/*−*^ mice were decreased after rapamycin treatment, which were similar to those in the control mice with rapamycin treatment (Fig. S11A to C, Additional file 11). However, protein levels of Ki67, GPR41, and GPR43 in *RhoB*^*−*/*−*^ mice are still significantly increased compared with those in the control mice after rapamycin treatment (Fig. S11D to F, Additional file 11). Altogether, these results suggested that RhoB expression leads to microbiome alteration via a mechanism independent of autophagy.

### The abundance of P. denticola and A. rava are dramatically elevated in RhoB^+/−^ and RhoB^−/−^ mice

**Fig. 7:**
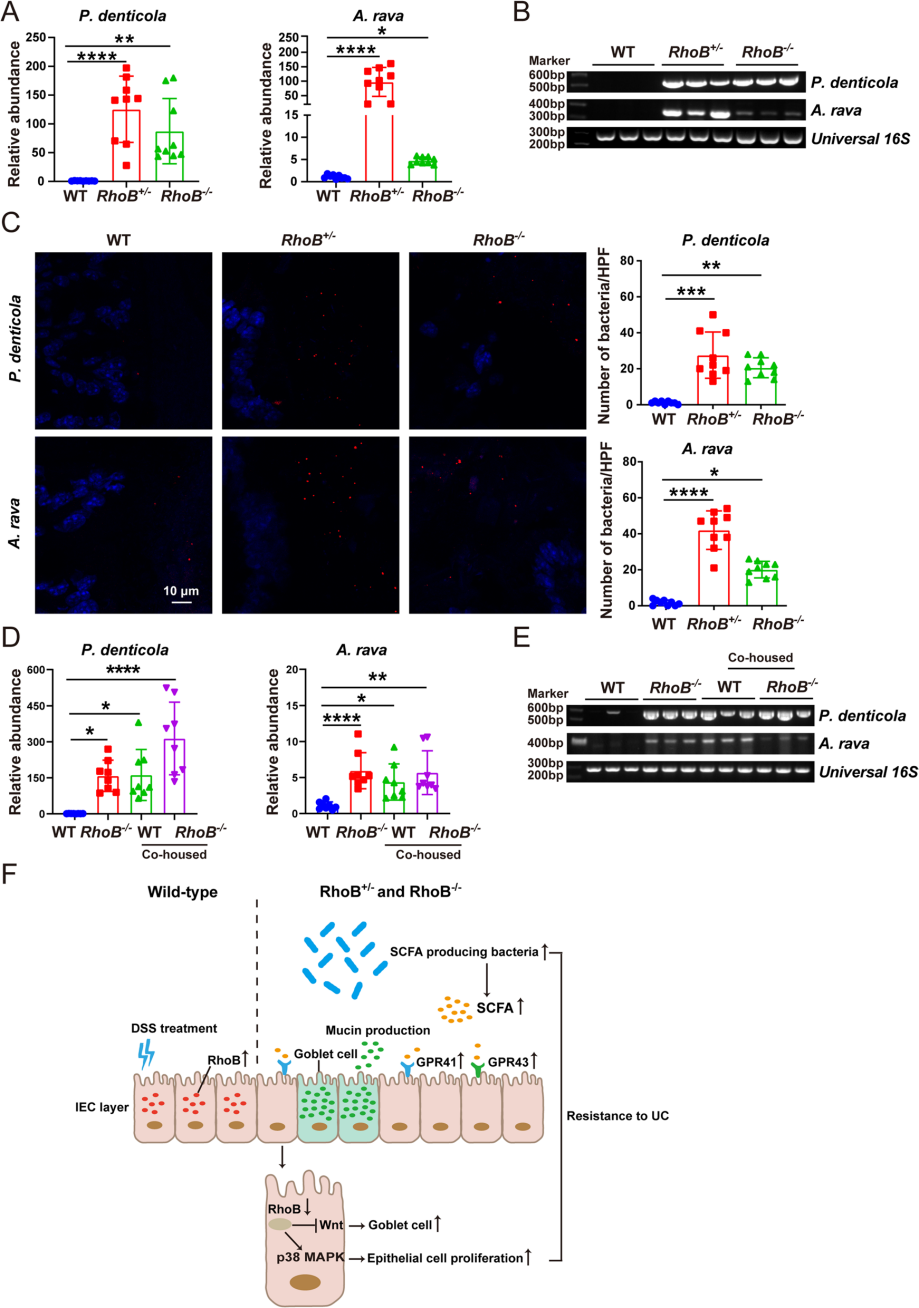
RhoB deficiency increases the abundance of *P. denticola* and *A. rava*. **A** RT-PCR analysis of the indicated 16S rRNA genes in the feces from WT and RhoB-deficient mice (*n* = 9 from 3 independent experiments). **B** DNA electrophoresis analysis of the indicated 16S rRNA genes in the feces from WT and RhoB-deficient mice (*n* = 3). **C** Representative confocal images of *P. denticola* and *A. rava* and quantitation in colon sections as indicated (*n* = 9 from 3 independent experiments). *P. denticola* or *A. rava*: Red; DAPI: blue. Scale bar: 10 μm. **D** RT-PCR analysis of the indicated 16S rRNA genes in the feces from WT and *RhoB*^*−/−*^ mice before and after cohousing (*n* = 8 from 2 independent experiments). **E** DNA electrophoresis analysis of the indicated 16S rRNA genes in the feces from WT and *RhoB*^*−/−*^ mice before and after cohousing (*n* = 3). **F** Graphical model illustrating the role of RhoB in colitis. Data are the mean ± SD. One-way ANOVA (**A**, **C**, and **D**). *p < 0.05, **p < 0.01, ***p < 0.001, ****p < 0.0001

We further analyzed operational taxonomic unit (OTU) representative sequences based on 16S rRNA gene sequencing to identify the species of *Prevotella* and *Alloprevotella* genus abundant in *RhoB*^*+/−*^ and *RhoB*^*−/−*^ mice. Comparison of 16S rRNA gene sequence indicated that *P. denticola* and *A. rava* seemed to be the representative species. We then measured the abundance of *P. denticola* and *A. rava* in *RhoB*^*+/−*^/*RhoB*^*−/−*^ and WT mice. Real-time PCR showed that the abundance of *P. denticola* and *A. rava* were upregulated in *RhoB*^*+/−*^ and *RhoB*^*−/−*^ mice, compared with that of WT mice (Fig. 7A). DNA electrophoresis further confirmed these changes (Fig. 7B). Additionally, the increased abundance of *P. denticola* and *A. rava* in *RhoB*^*+/−*^/*RhoB*^*−/−*^ mice were also identified by fluorescence in situ hybridization (FISH) (Fig. 7C). We next investigated the abundance of *P. denticola* and *A. rava* between WT and *RhoB*^*−/−*^ mice after cohousing. The abundance of *P. denticola* and *A. rava* in WT mice were similar to those of *RhoB*^*−/−*^ mice after cohousing, which were significantly increased compared with those in WT mice without cohousing (Fig. 7D, E). Taken together, these data suggest that the increased abundance of *P. denticola* and *A. rava* are associated with decreased colitis in RhoB-deficient mice.

## Discussion

In this study, we demonstrated that decreased RhoB exerts critical effects on maintaining the intestinal epithelial homeostasis. Interestingly, we noted that *RhoB*^+/*−*^ mice exhibited better effect on alleviating colitis symptoms than *RhoB*^*−*/*−*^ mice (Fig. 2A–F). *RhoB*^+/*−*^ mice had a higher relative abundance of *Prevotella* and *Alloprevotella* compared with *RhoB*^*−*/*−*^ mice. Consistently, the concentration of SCFAs and GPR41/43 in *RhoB*^+/*−*^ mice was higher than that of *RhoB*^*−*/*−*^ mice, whereas the number of Ki67^+^ cells and the Muc2 expression were lower in *RhoB*^+/*−*^ colonic epithelium and *RhoB*^+/*−*^ organoids compared to those in *RhoB*^*−*/*−*^ mice. Thus, the better effect of *RhoB*^+/*−*^ than *RhoB*^+/*−*^ in DSS-induced colitis is mainly a result from intestinal microbiome, indicating that RhoB-mediated intestinal microbiome alternation plays an important role in resistance to DSS-induced colitis.

It was reported that miR-21 overexpression impairs intestinal barrier, and miR-21 overexpression decreased RhoB expression, likely suggesting a protective role for RhoB in UC [27, 28]. However, the direct role of RhoB in intestinal barrier and UC were not examined. In our study, we showed that RhoB deficiency protected against colitis directly. In addition, these studies showed that RhoB expression was downregulated in a few colon tissues of UC patients. However, we noticed that UC patients included in these studies were mostly treated with antibiotics. Our current findings showed that antibiotics treatment diminished RhoB expression during colitis (Fig. 1F). Although speculative, it is possible that antibiotics treatment have contributed to the observation of decreased RhoB expression in these reports.

Our previous study identified a vital role of RhoB in promoting autophagy [19]. Therefore, we speculate that decreased RhoB may shape microbiota by regulating autophagy. However, rapamycin treatment to induce autophagy did not restore the decreased severity of colitis and increased SCFA-producing bacteria in mice, indicating that altered microbiota was not due to autophagy. Notably, we found that *RhoB*^+/*−*^ and *RhoB*^*−/−*^ mice exhibited enhanced epithelial barrier function including increased goblet cells, Muc2 protein level, and thickness of the inner mucus layer. Mucus layer is reported to modulate commensal and pathogenic bacterial microbiota [21, 29, 30]. Thus, it is possible that enhanced epithelial barrier function may lead to altered microbiota in *RhoB*^+/*−*^ and *RhoB*^*−/−*^ mice. However, further study is required to clarify the mechanism underlying the alteration of microbial composition in RhoB-deficient mice.

Several studies have shown that levels of fecal SCFAs, which are beneficial to human health by maintaining intestinal homeostasis, are reduced in patients with UC [31–33]. In the present study, we found that fecal SCFAs levels are increased in *RhoB*^+/*−*^ and *RhoB*^*−/−*^ mice, which is in accordance with the increased abundances of SCFA-producing microbiota including *Prevotella, Alloprevotella*, *Lachnoclostridium*, *Roseburia*, and *Odoribacter*. In addition, abundances of *Prevotella, Alloprevotella*, *Lachnoclostridium*, *Roseburia*, and *Odoribacter* are also reported to be decreased in DSS-induced colitis [22, 23, 34–36]. Moreover, some species of the genera *Prevotella* and *A. rava* are found to generate SCFAs and exert an anti-inflammatory effect in colitis [22, 23]. Consistent with this, the present study showed abundances of *P. denticola* and *A. rava* are dramatically elevated in *RhoB*^+/*−*^ and *RhoB*^*−/−*^ mice.

Results from our in vitro and in vivo studies suggested that decreased RhoB promotes goblet cell production and epithelial cell proliferation via repressing Wnt/β-catenin signaling and activating p38 MAPK signaling. Nevertheless, we noticed that mRNA levels of *Muc2* and transcription factors essential for goblet cell differentiation (*Hes5*, *Spdef*, and *Klf4*) were not increased in RhoB-deficient mice (data not shown). Since RhoB regulates multiple signaling pathways including the Egrf, Ras, PI3K/Akt/mTOR, and Myc [18], we speculate that RhoB may influence Muc2 and some goblet cell transcription factors through post-transcriptional modification by interfacing with intracellular signaling pathways. The exact mechanisms through which *RhoB*^*−/−*^ repressed Wnt/β-catenin signaling and activated p38 MAPK signaling can benefit from further study.

## Conclusions

This study demonstrated that RhoB was increased in colonic epithelia of patients with severe UC and mice with DSS-induced colitis, reduced level of RhoB provided benefit for DSS-colitis remission with increased goblet cells and IEC proliferation, and also induced beneficial intestinal microbiome for colitis remission in mice. It reveals that RhoB is an important factor in regulating intestinal microbiota and homeostasis, which suggests that RhoB could be used as a new biomarker for UC and provide a potential therapeutic target for UC treatment.

## Methods

### Mice

All mice were generated on the C57BL/6J background. RhoB heterozygous (*RhoB*^*+/−*^) and homozygous knockout (*RhoB*^*−/−*^) mice were generated at the Shanghai Biomodel Organisms Center (Shanghai, China) and were confirmed by PCR genotype analysis [19]. Wild type male C57BL/6J mice were purchased from Academy of Military Medical Science (Bejing, China). All mice were maintained in a specific pathogen free condition in the Department of Laboratory Animal Science and Technology of Tianjin Medical University. Unless otherwise stated, all mice were used for experiments at 6–8 weeks of age. All mice experiments were approved by Animal Care and Use Committee, Tianjin Medical University (TMUaMEC 2021039).

### Human samples

Human colonic biopsies were obtained from Tianjin Medical University General Hospital, Tianjin, China. The diagnosis of UC was based on colonoscopy and pathological examination of colonic biopsies. Clinical characteristics of patients with UC are shown in Table S1 (Additional file 12). All human biopsies were obtained with informed consent, and the study was approved by the Ethics Committee of General Hospital, Tianjin Medical University, China (IRB2020-KY-190).

### Cell lines and reagents

The human colorectal cancer cell line SW480 (ATCC ccl-228, RRID CVCL-0546) was obtained from ATCC. Cells were cultured in RPMI 1640 medium supplemented with 10% bovine fetal serum and incubated in a humidified atmosphere containing 5% CO_2_ at 37 °C. The p38/MAPK inhibitor (10 μM; SB239063, Med Chem Express, NJ, USA), the PI3K-Akt inhibitor (20 μM; S1078, Selleck Chemicals, Houston, TX, USA), and the Wnt activator (5 μM; S2924, Selleck Chemicals) were dissolved in DMSO (Sigma, St. Louis Missouri, USA) and added to the culture medium for 24 h.

### GEO datasets

For gene expression analysis, microarray data reported in the Gene Expression Omnibus (https://www.ncbi.nlm.nih.gov/geo/) (GSE97012, GSE59071, GSE48958 and GSE75214) were analyzed using Sanger Box software (http://sangerbox.com/).

### ScRNAseq analysis

Single-cell RNA sequencing (scRNAseq) data from colon biopsies of patients with UC were analyzed with the Broad Institute Single Cell portal (https://singlecell.broadinstitute.org/single_cell/study/SCP259/intra-and-inter-cellular-rewiring-of-the-human-colon-during-ulcerative-colitis). The RhoB expression analysis was performed by Majorbio Bio-Pharm Technology Co., Ltd. (Shanghai, China).

### Western blotting

Distal colon was homogenized in RIPA lysis buffer, supplemented with complete protease inhibitors (Roche, Mannheim, Germany). Protein lysates were boiled with 5 × SDS-buffer in 99 °C for 10 min. Samples were then separated on 10% SDS-PAGE gels and transferred to PVDF membrane. The PVDF membrane was blocked with 5% milk and incubated with anti-RhoB antibody (1:1000, Cell Signaling Technology, China) overnight in 4 °C, followed by incubation for 1 h with appropriate secondary antibodies. Protein bands were visualized with Immobilon Western Chemiluminescent HRP Substrate (Merck Millipore, Billerica, MA, USA). Densitometery was performed using ImageJ software (National Institutes of Health, Bethesda, USA).

### DSS-induced acute and chronic colitis mouse model

For DSS-induced acute colitis mouse model, mice were given 1.5 % DSS (0216011090, MV=36–50 kDa, MP, USA) dissolved in drinking water for 7 days. For DSS-induced chronic colitis mouse model, mice were given 1% DSS in drinking water for 7 days and followed by normal drinking water for an additional 7 days with 3 cycles of treatment. Weight, rectal bleeding, and stool consistency were monitored daily and scored as previously reported [37]. The disease activity index (DAI) of mice were scored according to body weight loss, rectal bleeding, and stool consistency. Body weight loss was determined as follows: 0 (<2%), 1 (2–5%), 2 (5–10%), 3 (10–15%), 4 (≥15%). Rectal bleeding was determined as follows: 0, normal; 1, brown color; 2, reddish color; 3, bloody stool. Stool consistency was determined as follows: 0, normal; 1, mild soft stools; 2, very soft stools; 3, watery stools [37]. On the seventh day, mice were euthanized, and colon tissues were collected. A distal part of colon was fixed in 4% paraformaldehyde and embedded into paraffin, followed by histological analysis. A distal part of colon was embedded in optimal cutting temperature (OCT) compound, immediately snap-frozen in liquid nitrogen, and then transferred to −80 °C for storage.

### Intestinal permeability assay

At day 6 after 1.5% DSS treatment, mice were gavaged orally with FITC-dextran (0.6 mg/g body weight, MW 3000–5000, Sigma-Aldrich, St. Louis, MO, USA). Blood was collected by cardiac puncture at 4 h after FITC-dextran administration. The blood was centrifuged at 4 °C 2000*g* for 10 min. Plasma was analyzed for measuring fluorescence intensity with Synergy HT Multi-Mode Microplate Reader (Bio-tek Winooski, Vermont, USA) at an excitation wavelength of 485 nm and emission wavelength of 525 nm.

### Cohousing experiment

Two male WT and two male *RhoB*^+/*−*^ or *RhoB*^*−*/*−*^ mice were cohoused in a single cage at the time of weaning (4 weeks of age) and maintained for 4 weeks. At least 3 cages were used for cohousing of WT and *RhoB*^+/*−*^ or *RhoB*^*−*/*−*^ mice. After cohousing, mice were treated with 1.5% DSS for 7 days and then colon tissues and fecal samples were collected.

### Rapamycin treatment

Mice were injected intraperitoneally with rapamycin (2.5 mg/kg body weight/day, HY-10219, Med Chem Express) for 7 days, at which time 1.5% DSS in drinking water was given and the rapamycin treatment was continued for additional 7 days. Feces samples were collected on the zero day and colon tissues were collected on the zero day and seventh day.

### RNA interference

Small interfering RNAs (siRNA) targeting RhoB and scrambled siRNA (siScr) were synthesized by GenePharma (Shanghai, China). The sequences of the siRNAs are listed in Table S2 (Additional file 13). SW480 cells were transfected with siRNA using Lipofectamine 3000 (Invitrogen, Carlsbad, CA, USA). Protein expression of the siRNA target was assessed using western blotting.

### Antibiotic treatment

Mice were gavaged with a cocktail of antibiotics (Abx, ampicillin (A610028-0025, 1 g/L), neomycin (A610366-0025, 1 g/L), metronidazole (A600633-0025, 1 g/L), and vancomycin (A600983-0001, 500 mg/L), SangonBiotech, Shanghai, China) in addition with Abx-containing drinking water for 7 days [38]. Abx-containing water was renewed every 3 to 4 days to maintain efficacy. Mice were treated with 1.5% DSS in drinking water for another 7 days and colon tissues were collected on the seventh day.

### Hematoxylin and eosin (H&E) staining

For histological analysis, distal colon tissue was fixed in 4% paraformaldehyde and embedded into paraffin. Tissue sections (5 μm) were stained with hematoxylin and eosin. Images were captured with a microscope (BX46, Olympus, Tokyo, Japan). Histological score was evaluated according to the criteria described before [37]. Briefly, histological score was scored with seven parameters: degree of inflammation (0–4), the severity of crypt damage (0–4), immune cell infiltration (0–3), submucosal edema (0–3), loss of goblet cells (0–3), active epithelial hyperplasia (0–3), and crypt abscesses (0–2).

For goblet cell detection, AB-PAS staining was performed with the AB-PAS Stain Kit (Solarbio, Beijing, China) according to the manufacturer’s recommendations. Images were captured with a microscope (BX46, Olympus). The total number of goblet cells was counted in crypts from 4 different fields.

### Immunohistochemistry

For immunohistochemical staining and imaging, tissue sections were deparaffinized, dehydrated, and subjected to antigen retrieval in citrate buffer (pH 6). The sections were then exposed to 0.3% hydrogen peroxide to block endogenous peroxidase activity, blocked with 5% normal goat serum for 1 h, and stained overnight with primary antibodies against Muc2 (1:1000, Proteintech, Chicago, IL, USA), Ki67 (1:50, Abcam, Cambridge, MA, USA), Lgr5 (1:400, Affinity Biosciences, Cincinnati, OH, USA ), Caspase3 (1:2000, Cell Signaling Technology, Danvers, MA, USA), ZO-1 (1:500, Thermo Fisher Scientific, Waltham, MA, USA), Occludin (1:200, Abcam), GPR41 (1:1000, Abcam), GPR43 (1:500, Abcam), β-catenin (1:200, Cell Signaling Technology), or p-p38 (1:200, Cell Signaling Technology). The sections were washed at least five times with PBS and stained for 30 min with horseradish peroxidase (HRP)-labeled secondary antibody (Zsbio, Beijing, China). The staining was developed with diaminobenzidine chromogenic substrate (Zsbio). The sections were finally counterstained with hematoxylin, dehydrated, and mounted. Tissues were imaged using an Olympus BX46 microscope. Staining intensity was analyzed using the software Image Pro Plus.

### IEC harvest

For RNA sequencing analyses, IEC were extracted from WT and *RhoB*^*−*/*−*^ mice as described previously [39]. Briefly, the colons were cut longitudinally, divided into 1-cm pieces, and incubated in ice-cold PBS containing 30 mM EDTA and 1.5 mM DTT for 30 min. The remaining colons were then suspended in PBS containing 30 mM EDTA at 37 °C for 10 min. After incubation, samples were shaken gently for 30 s to release epithelium from basement membrane. The remnant intestinal tissue was removed. IEC were centrifuged at 1000 rpm for 5 min at 4 °C. After washing once with ice-cold PBS at 4 °C, the IEC were lysed in Trizol reagent (Invitrogen) for RNA extraction.

### Immunofluorescent staining

Tissues embedded in OCT compounds were cut into 5-μm sections. The sections were fixed with cold acetone for 10 min at 4 °C and then blocked with 5% BSA for 1 h. Samples were then incubated overnight with the indicated primary antibodies: Muc2 (1:500, Proteintech), GPR41 (1:400, Abcam) and GPR43 (1:400, Abcam) , CA1(1:200, Proteintech), Lgr5 (1:400, Affinity Biosciences), CHGA (1:100, Abcam), RhoB (1:200, Santa Cruz, CA, USA). Samples were washed with PBS and incubated for 2 h with Alexa Fluor 488-labeled second antibody or Alexa Fluor 594-labeled second antibody (1:500, Proteintech). Following extensive washing, the sections were mounted with DAPI Fluoromount-G gum. Images of sections were obtained using a confocal fluorescence microscope (Leica TCS-SP8, Leica Microsystems, Germany).

SW480 cells were fixed with 4% paraformaldehyde for 15 min and then permeabilized with 0.25% TritonX-100 for 10 min. The cells were blocked with 5% normal goat serum for 1 h, and incubated overnight with primary antibodies against Muc2 (1:500, Proteintech) and Ki67 (1:250, Abcam), followed by a washing step and incubation with Alexa Fluor 488-labeled second antibody and DAPI. Fluorescence was detected using a fluorescence microscope (OLYMPUS IX73, OLYMPUS, Japan).

### DNA extraction and 16S rRNA gene sequencing

Fecal genomic DNA was extracted from each mouse using the Stool DNA Kit (D4015-02, Omega Bio-tek, Norcross, GA, USA) according to the manufacturer’s recommendations. The final DNA concentration and purification were determined by a NanoDrop 2000 UV-vis spectrophotometer (Thermo Scientific, Wilmington, USA). The DNA quality was checked by 1% agarose gel electrophoresis.

16S rRNA gene sequencing was performed by Majorbio Bio-Pharm Technology Co., Ltd. (Shanghai, China). The V3-V4 hypervariable regions of the bacteria 16S rRNA gene were amplified with the 515F/806R primer set by thermocycler PCR system (GeneAmp 9700, ABI, USA). The sequences of the 515F/806R primer set are listed in Table S2 (Additional file 13). The amplification was initiated with denaturation at 95 °C for 3 min, followed by 30 cycles at 95 °C for 30 s, 55 °C for 30 s, and 72 °C for 45 s, and a last extension at 72 °C for 5 min. The 20 μL reaction mixture contained 4 μL 5 × FastPfu Buffer, 0.25 mM dNTPs, 200 nM 515F primer, 200 nM 806R primer, 2 units of FastPfu Polymerase, and 10 ng template DNA. The PCR products were extracted from a 2% agarose gel and further purified using the AxyPrep DNA Gel Extraction Kit (Axygen Biosciences, Union City, CA, USA). The PCR products were quantified using QuantiFluor™-ST (Promega, USA) according to the manufacturer’s protocol.

Purified amplicons were pooled in equimolar and paired-end sequenced (2 × 300) on an Illumina MiSeq platform (Illumina, San Diego, USA) according to the standard protocols by Majorbio Bio-Pharm Technology Co. Ltd. (Shanghai, China). Raw fastq files were quality-filtered by Trimmomatic and merged by FLASH. Operational taxonomic units (3OTUs) were clustered with 97% similarity cutoff using UPARSE (version 7.1 http://drive5.com/uparse/) with a novel “greedy” algorithm that performs chimera filtering and OTU clustering simultaneously. The taxonomy of each 16S rRNA gene sequence was analyzed by RDP Classifier algorithm (http://rdp.cme.msu.edu/) against the Silva (SSU123) 16S rRNA database using confidence threshold of 70%. Data analysis of alpha diversity, beta diversity, bacterial taxonomic distributions, and generation of the heatmap were performed at Majorbio I-Sanger Cloud platform (https://cloud.majorbio.com). The raw reads were deposited into the NCBI Sequence Read Archive (SRA) database (Accession Number: SRP312259, SRP312236, and SRP312057).

### RNA sequencing

Total RNA was extracted from tissues using TRIzol Reagent according the manufacturer’s instructions (Invitrogen). The RNA quality was determined using a 2100 Bioanalyser (Agilent), and the RNA concentration was quantified using the ND-2000 (NanoDrop Technologies). RNAseq transcriptome library was prepared following TruSeqTM RNA sample preparation Kit from Illumina (San Diego, CA) using 1 μg of total RNA. Paired-end RNAseq sequencing library was sequenced with the Illumina HiSeq xten/NovaSeq 6000 sequencer (2×150 bp read length) according to the standard protocols by Majorbio Bio-Pharm Technology Co. Ltd.

The raw paired-end reads were trimmed and quality controlled by SeqPrep (https://github.com/jstjohn/SeqPrep) and Sickle (https://github.com/najoshi/sickle) with default parameters. Then clean reads were separately aligned to reference genome with orientation mode using HISAT2 software (http://ccb.jhu.edu/software/hisat2/index.shtml). The mapped reads of each sample were assembled by StringTie (https://ccb.jhu.edu/software/stringtie/index.shtml?t=example) in a reference-based approach.

To identify DEGs (differential expression genes) between two different samples, the expression level of each transcript was calculated according to the transcripts per million reads (TPM) method. RSEM (http://deweylab.biostat.wisc.edu/rsem/) was used to quantify gene abundances. Analysis of differential gene expression, KEGG pathway, and GSEA were performed at Majorbio I-Sanger Cloud platform (https://cloud.majorbio.com). Detailed RNA sequencing data have been deposited in NCBI’s Gene Expression Omnibus (GEO) and are accessible through GEO series accession number GSE170994.

### SCFAs analysis

Concentrations of SCFAs were determined by GC-MS. GC-MS was performed with an Agilent 8890B GC and an Agilent 5977B mass analyzer (Agilent Technologies Inc. CA, UAS) according to the standard protocols by Majorbio Bio-Pharm Technology Co. Ltd. Quantification of SCFAs was calculated with external standard curve method and were normalized to sample weight.

### Real-time PCR

Fecal genomic DNA was extracted from each mouse using the Stool DNA Kit (Omega Bio-tek). The DNA concentration was determined using a NanoDrop 2000 UV-vis spectrophotometer (Thermo Scientific). The abundance of the indicated bacteria was analyzed with bacterial-specific primers by thermocycler PCR system (LightCyler 96, Roche). The sequences of the bacterial-specific primers are listed in Table S2 (Additional file 13). The 20 μl reaction mixture contained 20 ng DNA, 10 μM forward and reverse primers, and 1× Syber green (Cwbio, Beijing, China). The PCR program was as follows: 95 °C for 10 min, 35 cycles at 95 °C for 15 s, 52 °C for 60 s, 72 °C for 30 s. The relative abundance was calculated by comparing the cycle threshold values using the 2^*−*ΔΔCycle threshold^ method.

RNA from colonic tissues was extracted using the TRizol reagent (Solarbio, Beijing, China) in accordance with the manufacturer’s instructions. Two micrograms total RNA were reverse-transcribed to cDNA by using HiFiScript cDNA Synthesis Kit (Cwbio). After reverse transcription, real-time PCR amplification was performed using SYBR Green qPCR Master mixes (Cwbio) under thermocycler PCR system (LightCyler 96, Roche). The PCR program was as follows: 95 °C for 10 min; 35 cycles of 95 °C for 15 s, 60 °C for 30 s, and 72 °C for 30 s. 2^−ΔΔCT^ method was used to calculate the relative gene expression. β-Actin was used as an internal control. Sequences of the primer used are listed in Table S2 (Additional file 13).

### Culture and ICC of intestinal organoids

Isolation of crypt cells and organoid cultures were performed as described previously [40]. Briefly, the colon was cut longitudinally and washed with cold PBS. Crypts were incubated in Gentle Cell Dissociation Reagent (STEMCELL Technologies, Seattle, WA, USA) at room temperature on a rocking platform at 20 rpm for 20 min. Subsequently, crypts were filtered through a 70-μm cell strainer. The process was typically repeated four times. The crypt fraction was enriched by centrifugation at 290*g* for 5 min at 4 °C. The crypt pellets were resuspended in cold PBS with 0.1% BSA (w/v), transferred into a new tube, and then centrifuged at 200*g* for 3 min at 4 °C. The crypt pellets were resuspended in cold DMEM/F-12 with 15 mM HEPES (STEMCELL Technologies) and centrifuged at 200*g* for 3 min at 4 °C for final collection. Crypts were subsequently embedded with Matrigel (BD Biosciences, San Jose, CA, USA) on ice and plated in 24-well plates. After polymerization of Matrigel, 750 μL of complete IntestiCult™ Organoid Growth Medium (STEMCELL Technologies) at room temperature was added to each well. The culture medium was refreshed every 2 days. To confirm the role of Wnt and p38 MAPK signaling pathway in goblet cell differentiation and epithelial cells proliferation, organoids were cultured in culture medium with or without the p38/MAPK inhibitor (10 μM; SB239063, Med Chem Express) and the Wnt activator (5 μM; S2924, Selleck Chemicals) for 4 days.

For immunofluorescent staining, colonic organoids were seeded on glass coverslips in a 24-well culture plate. Organoids were fixed with cold 4% PFA at room temperature for 30 min, permeabilized with 0.2% Triton X-100 in PBS for 30 min, and blocked with 5% BSA for 1 h. Organoids were incubated overnight with primary antibodies against Muc2 (1:500, Proteintech) or Ki67 (1:250, Abcam). Organoids were washed with PBS and stained with Alexa Fluor 594-labeled second antibody at room temperature for 2 h. The coverslip was removed from the well and sided down onto the DAPI Fluoromount-G gum droplet on a glass slide. Images of organoids were taken by a confocal fluorescence microscope (Leica TCS-SP8, Leica Microsystems).

### Fecal microbiota transplantation (FMT)

Two hundred milligrams of stool was daily collected from WT donor mice or RhoB-deficient donor mice and resuspended in 1 mL sterile anaerobic PBS, and then centrifuged at 800×*g* for 3 min. The supernatant then passed through a 70-μm filter to remove large particulate. WT recipient mice were pretreated with broad spectrum antibiotic for 7 days and then were intragastrically administered with 0.2 mL fresh fecal solution from WT donor mice or RhoB-deficient donor mice once daily for 5 days. Thereafter, WT recipient mice was treated with 1.5% DSS for 7 days.

### Bone marrow transplantation

WT and RhoB-deficient recipient mice (expressing CD45.2 leukocyte antigen) was irradiated with 5 Gy. Bone marrows were collected with cold PBS from the femur and tibia of congenic WT (expressing CD45.1 leukocyte antigen). After several red blood cells (RBC) lysis, cells were resuspended with cold PBS. One day after irradiation, 1×10^7^ cells per mouse in 100 μL cold PBS was intravenously injected in irradiated recipient mice. After transplantation, recipient mice were treated with antibiotics-containing drinking water for 7 days. Antibiotics-containing water was renewed every 3 to 4 days to maintain efficacy. Bone marrow reconstitution was verified after 4 weeks by staining for CD45.1 and CD45.2 in peripheral blood cells. At 5 weeks after bone marrow transplantation, colon tissues were collected.

### Microbial FISH analysis

To evaluate the abundance of *P. denticola* and *A. rava* in RhoB-deficient and WT mice, fluorescence in situ hybridization (FISH) was performed on colon containing stool using DNA bacterial universal FISH Kit (D-0016, Exonbio, Guangzhou, China). 16S rRNA-targeted oligonucleotide probes were synthesized by Exonbio (Guangzhou, China). The sequences of probes are listed in Table S2 (Additional file 13). Images were obtained using a confocal fluorescence microscope (Leica TCS-SP8, Leica Microsystems).

### Isolation of mesenteric lymph nodes (mLN) and colonic lamina propria cells

To isolate lymphocytes from the mLN, the nodes were ground directly with RPMI 1640 medium supplemented with 10% FBS using a 70-μm cell strainer. After centrifugation, the cells were resuspended in RPMI 1640 medium for evaluation.

To isolate lymphocytes from the colonic lamina propria, the colon tissues were cut into 1-cm pieces in cold PBS and incubated with cold PBS containing 2 mM dithiothreitol (DTT) at 150 rpm and 37 °C for 10 min, followed by incubating with cold PBS containing 5 mM EDTA three times at 150 rpm and 37 °C for 10 min. Next, the colon tissue was minced with scissors in 1 mL digestion solution (0.5 mg/mL collagenase (Sigma-Aldrich), 0.05 mg/mL hyaluronic acid, and 100 ng/mL DNase I in RPMI 1640 medium) at 150 rpm and 37 °C for 30 min. After incubation, the cell solution was strained through a 70-μm cell strainer and suspended in 40% Percoll (Healthcare, Chicago, IL, USA), followed by centrifugation at 670*g* for 30 min at 4 °C. The cell pellet was washed with cold PBS and resuspended in RPMI 1640 medium for evaluation.

### Flow cytometry

For cell surface staining, single-cell suspensions were stained for 30 min at 4 °C in the dark with the following antibodies: anti-CD45 conjugated to APC (1:100, 103111, Biolegend, San Diego, CA, USA), anti-CD11b conjugated to PE-Cy7 (1:100, 25-0112-81, Invitrogen), anti-F4/80 conjugated to FITC (1:100, 11-4801-85, eBioscience, San Diego, CA, USA), anti-CD45 conjugated to APC-Cy7 (1:100, 103116, Biolegend), anti-MHCII conjugated to PE (1:100, 107607, Biolegend), anti-CD11c conjugated to APC (1:100, 117309, Biolegend), anti-CD45 conjugated to PerCP/Cy5.5 (1:100, 103132, Biolegend), anti-Ly6G conjugated to APC (1:100, 127614, Biolegend). For intracellular cytokine staining, single-cell suspensions were stimulated for 5 h at 37 °C with cell activation cocktail with Brefeldin A (1:100, 423303, Biolegend). After this period, cells were washed with PBS and stained for cell viability with Zombie NIRTMFixable Viability Kit (1:1000, 423105, Biolegend) for 30 min at 4 °C, followed by staining with the following antibodies: anti-CD3 conjugated to FITC (1:100, 100204, Biolegend) and anti-CD4 conjugated to PE-Cy7 (1:100, 100422, Biolegend). Then, cells were fixed in fixation buffer (420801, Biolegend) and permeabilized with intracellular staining permeabilization wash buffer (421002, Biolegend) according to the manufacturer’s instructions. Cells were stained for 30 min at 4 °C with the following antibodies: anti-IL-17A conjugated to APC (1:100, 509616, Biolegend), anti-IFN-γ conjugated to PerCP/Cy5.5 (1:100, 45-7311-82, Invitrogen), anti-IL-4 conjugated to PE (1:100, 504103, Biolegend). For Treg cell analysis, cells were stained with the following antibodies: anti-CD3 conjugated to FITC (1:100, 100204, Biolegend), anti-CD4 conjugated to PE-Cy7 (1:100, 100422, Biolegend), and anti-CD25 conjugated to PE (1:100, 102008, Biolegend). Then, cells were fixed and permeabilized with Foxp3/transcription factor staining buffer sets (00-5523-00, eBioscience) according to the manufacturer’s instructions, followed by staining with anti-Foxp3 conjugated to APC (1:100, 320014, Biolegend). The data was acquired on FACS Canto II Flow Cytometer (BD Biosciences) and was analyzed with the FlowJo software (FlowJo, Ashland, OR, USA).

### Statistical analysis

Data were presented as the mean ± SD. The statistical significance was determined by unpaired Student’s *t* test, one-way ANOVA, or two-way ANOVA. Pearson correlation was used to analyze the correlation. Statistical analyses were performed using GraphPad Prism 6 (San Diego, CA). *p* < 0.05 was considered as a statistically significant difference.

## Supplementary Information

**Additional file 1: Figure S1**. RhoB was significantly increased in epithelial cells in colon biopsies of UC patients. (A-I) RhoB scRNAseq analysis in colon tissues of UC patients. (A) RhoB visualised on the single-cell RNAseq of human colon cells of UC patients in Epithelial tSNE. (B) Violin plot indicating the expression of RhoB in epithelial cells from colon biopsies of UC patients and healthy controls. (C) Violin plot indicating the average expression of RhoB in epithelial cells from colon biopsies of UC patients and healthy controls. (D) RhoB visualised on the single-cell RNAseq of human colon cells of UC patients in Immune tSNE. (E) Violin plot indicating the expression of RhoB in immune cells from colon biopsies of UC patients and healthy controls. (F) Violin plot indicating the average expression of RhoB in immune cells from colon biopsies of UC patients and healthy controls. (G) tSNE plot visualizing the annotation and color codes for cell subset in epithelial cell from colon biopsies of UC patients and healthy controls. (H) tSNE plots highlighting the expression of RhoB in epithelial cell from colon biopsies of UC patients and healthy controls. (I) Violin plot indicating the expression of RhoB in the different clusters from colon biopsies of UC patients and healthy controls. (J) Representative confocal images of CA1, Muc2, Lgr5, CHGA, and RhoB staining in colonic tissues of the DSS-treated wild type mice. CA or Muc2 or Lgr5 or CHGA: red; RhoB: Green; DAPI: blue. Scale bar: 10 μm or 5μm. (K) Western blotting analysis of RhoB expression in colonic tissues of indicated genotypes. β-Actin serves as a loading control. (L) Immunohistochemistry analysis of RhoB expression in colonic tissues of the indicated genotypes. Scale bar: 50 μm.

**Additional file 2: Figure S2**. RhoB deficiency alleviates inflammatory responses to DSS treatment. (A) mRNA expression levels of anti-microbial peptides and proinflammatory cytokines in the colons of indicated mice measured by real-time PCR (*n* = 9 from 2 independent experiments). (B) Gating strategy for flow cytometry analysis of immune cells. (C) Representative flow cytometry analysis of the indicated cells (left) and percentage (right) of indicated cells in mesenteric lymph nodes (MLN) and colonic lamina propria (cLP) from indicated naive mice (*n* = 9 from 2 independent experiments). (D) Representative flow cytometry analysis of the indicated cells (left) and percentage (right) of indicated cells in MLN and cLP from indicated mice after DSS administration (*n* = 9 from 2 independent experiments). Data are the mean ± SD. Statistical significance was determined by one-way ANOVA (A, C and D). *p < 0.05, **p < 0.01, ***p < 0.001.

**Additional file 3: Figure S3**. RhoB deficiency protects mice from DSS-induced chronic colitis. (A-F) Eight-week-old male mice were given 1% DSS in drinking water for seven days and followed by normal drinking water for an additional 7 days with 3 cycles of treatment (*n* = 9 from 2 independent experiments). (A) Schematic image illustrating chronic colitis design. (B) Body weight loss of the indicated mice and treatments. (C) Disease activity index of the indicated mice and treatments. (D) Measurement and quantification of colon length in the indicated mice. (E) Representative H&E staining analysis of histopathological changes and quantitation of histology score in colon from the indicated mice. (F) Alcian blue-Periodic acid Schiff (AB-PAS; indicating goblet cells) staining in colon from the indicated mice and treatments. Scale bar: 50 μm. (G) Measurement and quantification of colon length in naïve WT, *RhoB*^*+/-*^, and *RhoB*^*-/-*^ mice (*n* = 9 from 3 independent experiments). (H) Representative H&E staining analysis of histopathological changes in colon from naïve WT, *RhoB*^*+/-*^ and *RhoB*^*-/-*^ mice. (I) Representative Lgr5, Occludin, ZO-1, and cleaved caspase-3 staining and quantitation in colon sections as indicated (*n* = 9 from 3 independent experiments). Scale bar: 50 μm. Data are the mean ± SD. Statistical significance was determined by two-way ANOVA (B and C) or Unpaired Student’s t-test (D-F) or one-way ANOVA (G and I). **p < 0.01, ***p < 0.001, ****p < 0.0001. NS, not significant.

**Additional file 4: Figure S4**. SB239063 and CHIR99021 could respectively restore increased expression of Ki67 and Muc2 in siRhoB transfected SW480 cells. (A) Western blotting analysis of *RhoB* knockdown efficiency in SW480 cells as indicated. β-Actin serves as a loading control. (B) Representative confocal images of Muc2 staining (red) and DAPI (blue) in SW480 cells and quantification. (C) Representative confocal images of Ki67 staining (green) and DAPI (blue) in SW480 cells and quantification. (D) Representative confocal images of Ki67 staining (green) and DAPI (blue) and quantification in SW480 cells as indicated. (E) Representative confocal images of Muc2 staining (red) and DAPI (blue) and quantification in SW480 cells as indicated. Scale bar: 100 μm (B-E). *n* = 3 from 3 independent experiments (B-E). Data are the mean ± SD. Unpaired Student’s t-test (B-C) or one-way ANOVA (D-E). **p < 0.01, ***p < 0.001, ****p < 0.0001. NS, not significant.

**Additional file 5: Figure S5**. Altered microbiota in *RhoB*^*+/-*^ and *RhoB*^*-/-*^ mice. Stool samples from 6-8 week old WT, *RhoB*^*+/-*^ or *RhoB*^*-/-*^ mice were collected and analyzed by 16S rRNA gene sequencing (*n* = 5). (A) The amounts of bacterial DNA. (B) Analysis of the Shannon diversity index of microbiota. (C) PCoA results based on unweighted UniFrac distances. (D) Relative abundance of bacteria by taxon-based analyses. (E) Heatmap to visualize the relative abundances of the 30 most predominant bacterial genera.

**Additional file 6: Figure S6**. SCFA concentrations are positively correlated to the relative abundance of *Prevotella* and *Alloprevotella*. (A) Pearson’s correlation between the relative abundance of *Prevotella* and SCFAs in fecal from WT, *RhoB*^*+/-*^, and *RhoB*^*-/-*^ mice (*n* = 15). (B) Pearson’s correlation between the relative abundance of *Alloprevotella* and SCFAs in fecal from WT, *RhoB*^*+/-*^, and *RhoB*^*-/-*^ mice (*n* = 15). (C-F) Abx treatment eliminates the difference in the colitis phenotype of WT and RhoB-deficient mice (*n* = 5). (C) Scheme of Abx treatment protocol. (D) Disease activity index of WT and *RhoB*^+/-^ mice. (E) Measurement and quantification of colon length in WT and *RhoB*^+/-^ mice. (F) Representative H&E staining analysis of histopathological changes and quantitation of histology score in colons of WT and *RhoB*^+/-^ mice. Boxed areas in colon sections are enlarged and shown at the bottom panel. Scale bar: 200 μm or 50 μm. Data are the mean ± SD. Significance is determined using linear regression (A-B) or two-way ANOVA (D) or unpaired Student’s t-test (E and F). NS, not significant.

**Additional file 7: Figure S7**. The microbiota composition in the feces from WT mice shifts toward that of RhoB-deficient mice after cohousing. Stool samples from WT and RhoB-deficient mice before and after cohousing were collected and analyzed by 16S rRNA gene sequencing (*n* = 4). (A) Analysis of the Shannon diversity index of microbiota in the indicated genotypes. (B) PCoA analysis of microbiota. (C) Relative abundance of fecal microbiota at the genus level. (D) LEfSe analysis of distinctive microbiota composition as indicated.

**Additional file 8: Figure S8**. Intestinal microbiota does not affect goblet cell numbers, Muc2 and Ki67 protein levels. (A-C) WT and *RhoB*^-/-^ mice were cohoused for 4 weeks (*n* = 8 from 2 independent experiments). (A) Representative GPR41 and GPR43 staining and quantitation in colon sections as indicated. Scale bar: 50 μm. (B) Representative AB-PAS staining and quantification in colon sections of the indicated genotypes. (C) Representative Muc2 and Ki67 staining and quantitation in colon sections as indicated. (D-F) Autophagy induced by rapamycin does not reverse the colitis phenotype in *RhoB*^-/-^ mice. Mice were treated with rapamycin for 7 days and then treated with 1.5% DSS (*n* = 4 or 5). (D) Disease activity index of the indicated genotypes. (E) Measurement and quantification of colon length in the indicated genotypes. (F) Histopathological changes and quantitation of histology score in colon of the indicated genotypes. Scale bar: 50 μm. Data are the mean ± SD. Unpaired Student’s t-test (A to C) or two-way ANOVA (D) or one-way ANOVA (E-F). *p < 0.05, **p < 0.01, ***p < 0.001. NS, not significant.

**Additional file 9: Figure S9**. The effect of RhoB on intestinal phenotype depends on its expression in epithelia. CD45.1 WT bone marrow were transferred into irradiated CD45.2 WT, *RhoB*^*+/-*^ or *RhoB*^*-/-*^ mice respectively (*n* = 6 from 2 independent experiments) (A) Scheme of bone marrow transplantation protocol. (B) Flow cytometry analysis of CD45.1 expression in peripheral blood cells after bone marrow transplantation. The percentage of CD45.1^+^cells in lymphocyte cells from the indicated genotypes. (C) Representative H&E staining analysis of histopathological changes in colon of the indicated genotypes. (D) Representative AB-PAS staining and quantification in colon sections of the indicated genotypes. (E) Representative confocal images and quantitation of Muc2 staining (green) and DAPI (blue) in colonic tissues of the indicated genotypes. (F) Ki67 staining in colon sections of the indicated genotypes. (G) Representative GPR41 and GPR43 staining and quantitation in colon sections as indicated. Scale bar: 50 μm or 10 μm. Data are the mean ± SD. One-way ANOVA (B-G). *p < 0.05, **p < 0.01, ***p < 0.001, ****p < 0.0001.

**Additional file 10: Figure S10**. Autophagy does not contribute to microbiota alteration in *RhoB*^*-/-*^ mice. Stool samples from WT and *RhoB*^*-/-*^ mice before and after rapamycin treatment were collected and analyzed by 16S rRNA gene sequencing (*n* = 5). (A) Analysis of the Shannon diversity index of microbiota. (B) PCoA of microbiota. (C) Relative abundance of fecal microbiota at the genus level. (D) LEfSe analysis of distinctive microbiota composition as indicated. (E) Heatmap to visualize the relative abundances of the 30 most predominant bacterial genera.

**Additional file 11: Figure S11**. Autophagy does not affect Ki67, GPR41 and GPR43 protein levels. Mice were treated with rapamycin for 7 days and then were analyzed (*n* = 8 from 2 independent experiments). (A) Representative AB-PAS staining and quantification in colonic tissues of the indicated genotypes. (B) Representative Muc2 staining and quantitation in colon sections as indicated. (C) Representative confocal images of Muc2 staining in colonic tissues of the indicated genotypes. Quantification of the inner mucus layer thickness measured from the top of the villi to the periphery of the Muc2-positive region. Muc2: green; DAPI: blue. (D) Representative Ki67 staining and quantitation in colon sections as indicated. (E) Representative GPR41 staining and quantitation in colon sections as indicated. (F) Representative GPR43 staining and quantitation in colon sections as indicated. Scale bar: 10, 50 or 200 μm. Data are the mean ± SD. One-way ANOVA. ****p < 0.0001. NS, not significant.

**Additional file 12: Table S1**. Characteristics of Patients with UC and Healthy Controls.

**Additional file 13: Table S2**. Primers used in this study.

## Data Availability

All data needed to evaluate the conclusions in the paper are present in the paper and/or the supplementary materials. Additional data related to this paper may be requested from the authors. 16S rRNA gene sequencing data have been deposited into the NCBI Sequence Read Archive (SRA) database with the accession numbers SRP312259, SRP312236, and SRP312057. RNA sequencing data have been deposited in NCBI’s Gene Expression Omnibus (GEO) with the accession number GSE170994.
