# Supplementary material for: RhoB affects colitis through modulating cell signaling and intestinal microbiome
Source: Microbiome. 2022 Sep 16;10:149. doi: 10.1186/s40168-022-01347-3 (PMC9482252; doi:10.1186/s40168-022-01347-3)
Supplement: Supplementary file 2 — Additional file 1: Figure S1. RhoB was significantly increased in epithelial cells in colon biopsies of UC patients. (A-I) RhoB scRNAseq analysis in colon tissues of UC patients. (A) RhoB visualised on the single-cell RNAseq of human colon cells of UC patients in Epithelial tSNE. (B) Violin plot indicating the expression of RhoB in epithelial cells from colon biopsies of UC patients and healthy controls. (C) Violin plot indicating the average expression of RhoB in epithelial cells from colon biopsies of UC patients and healthy controls. (D) RhoB visualised on the single-cell RNAseq of human colon cells of UC patients in Immune tSNE. (E) Violin plot indicating the expression of RhoB in immune cells from colon biopsies of UC patients and healthy controls. (F) Violin plot indicating the average expression of RhoB in immune cells from colon biopsies of UC patients and healthy controls. (G) tSNE plot visualizing the annotation and color codes for cell subset in epithelial cell from colon biopsies of UC patients and healthy controls. (H) tSNE plots highlighting the expression of RhoB in epithelial cell from colon biopsies of UC patients and healthy controls. (I) Violin plot indicating the expression of RhoB in the different clusters from colon biopsies of UC patients and healthy controls. (J) Representative confocal images of CA1, Muc2, Lgr5, CHGA, and RhoB staining in colonic tissues of the DSS-treated wild type mice. CA or Muc2 or Lgr5 or CHGA: red; RhoB: Green; DAPI: blue. Scale bar: 10 μm or 5μm. (K) Western blotting analysis of RhoB expression in colonic tissues of indicated genotypes. β-Actin serves as a loading control. (L) Immunohistochemistry analysis of RhoB expression in colonic tissues of the indicated genotypes. Scale bar: 50 μm. [file 40168_2022_1347_MOESM1_ESM.pdf]

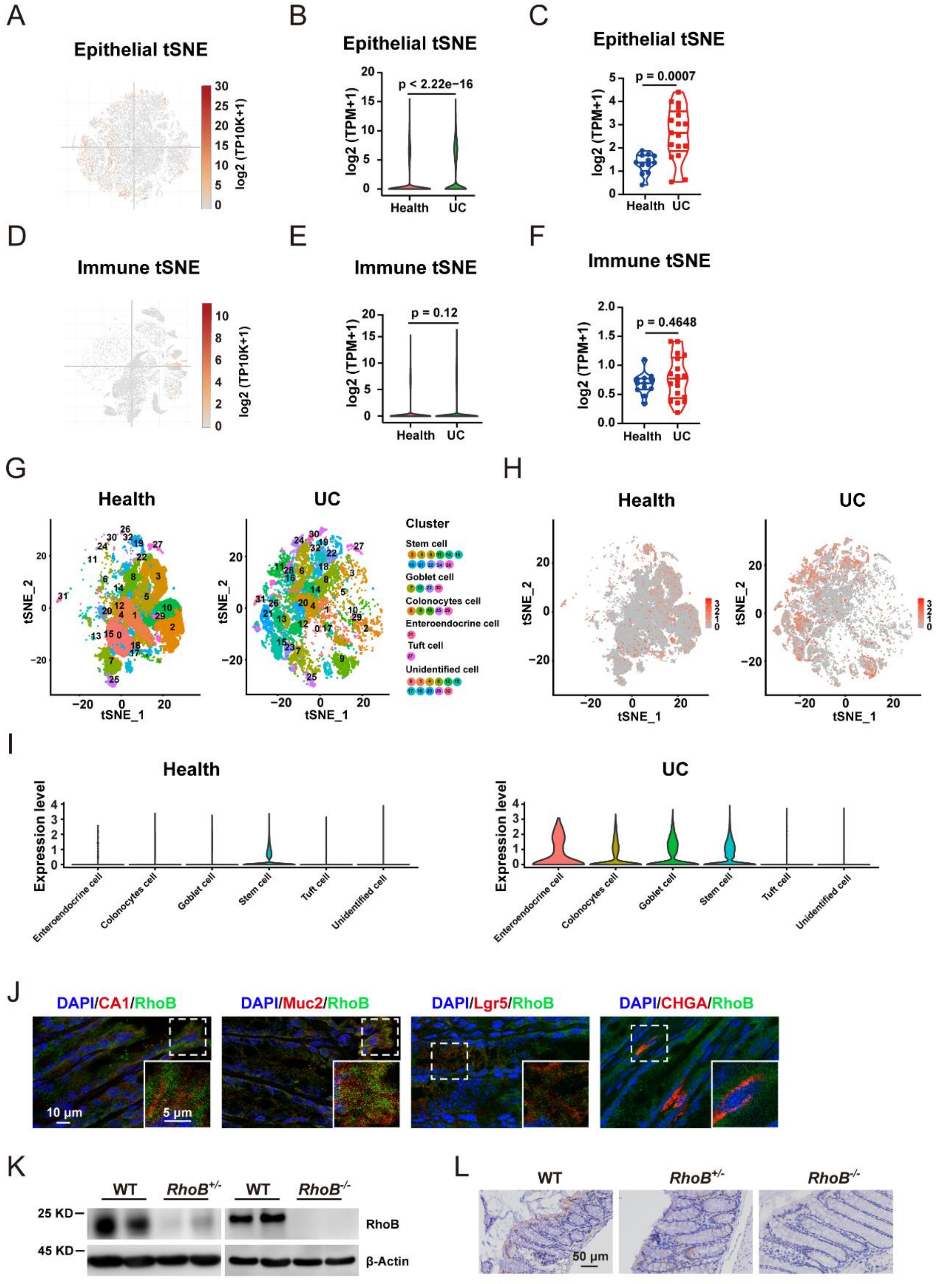

Figure S1. RhoB was significantly increased in epithelial cells in colon biopsies of UC patients. (A-I) RhoB scRNA-seq analysis in colon tissues of UC patients. (**A**) RhoB visualised on the single cell RNAseq of human colon cells of UC patients in Epithelial tSNE. (**B**) Violin plot indicating the expression of RhoB in epithelial cells from colon biopsies of UC patients and healthy controls. (**C**) Violin plot indicating the average expression of RhoB in epithelial cells from colon biopsies of UC patients and

healthy controls. (**D**) RhoB visualised on the single cell RNAseq of human colon cells of UC patients in Immune tSNE. (**E**) Violin plot indicating the expression of RhoB in immune cells from colon biopsies of UC patients and healthy controls. (**F**) Violin plot indicating the average expression of RhoB in immune cells from colon biopsies of UC patients and healthy controls. **(G)** tSNE plot visualizing the annotation and color codes for cell subset in epithelial cell from colon biopsies of UC patients and healthy controls. **(H)** tSNE plots highlighting the expression of RhoB in epithelial cell from colon biopsies of UC patients and healthy controls. **(I)** Violin plot indicating the expression of RhoB in the different clusters from colon biopsies of UC patients and healthy controls. **(J)** Representative confocal images of CA1, Muc2, Lgr5, CHGA, and RhoB staining in colonic tissues of the DSS-treated wild type mice. CA or Muc2 or Lgr5 or CHGA: red; RhoB: Green; DAPI: blue. Scale bar: 10 μm or 5μm. **(K)** Western blotting analysis of RhoB expression in colonic tissues of indicated genotypes. β-Actin serves as a loading control. **(L)** Immunohistochemistry analysis of RhoB expression in colonic tissues of the indicated genotypes. Scale bar: 50 μm.
