# Supplementary material for: RhoB affects colitis through modulating cell signaling and intestinal microbiome
Source: Microbiome. 2022 Sep 16;10:149. doi: 10.1186/s40168-022-01347-3 (PMC9482252; doi:10.1186/s40168-022-01347-3)
Supplement: Supplementary file 3 — Additional file 2: Figure S2. RhoB deficiency alleviates inflammatory responses to DSS treatment. (A) mRNA expression levels of anti-microbial peptides and proinflammatory cytokines in the colons of indicated mice measured by real-time PCR (n = 9 from 2 independent experiments). (B) Gating strategy for flow cytometry analysis of immune cells. (C) Representative flow cytometry analysis of the indicated cells (left) and percentage (right) of indicated cells in mesenteric lymph nodes (MLN) and colonic lamina propria (cLP) from indicated naive mice (n = 9 from 2 independent experiments). (D) Representative flow cytometry analysis of the indicated cells (left) and percentage (right) of indicated cells in MLN and cLP from indicated mice after DSS administration (n = 9 from 2 independent experiments). Data are the mean ± SD. Statistical significance was determined by one-way ANOVA (A, C and D). *p < 0.05, **p < 0.01, ***p < 0.001. [file 40168_2022_1347_MOESM2_ESM.pdf]

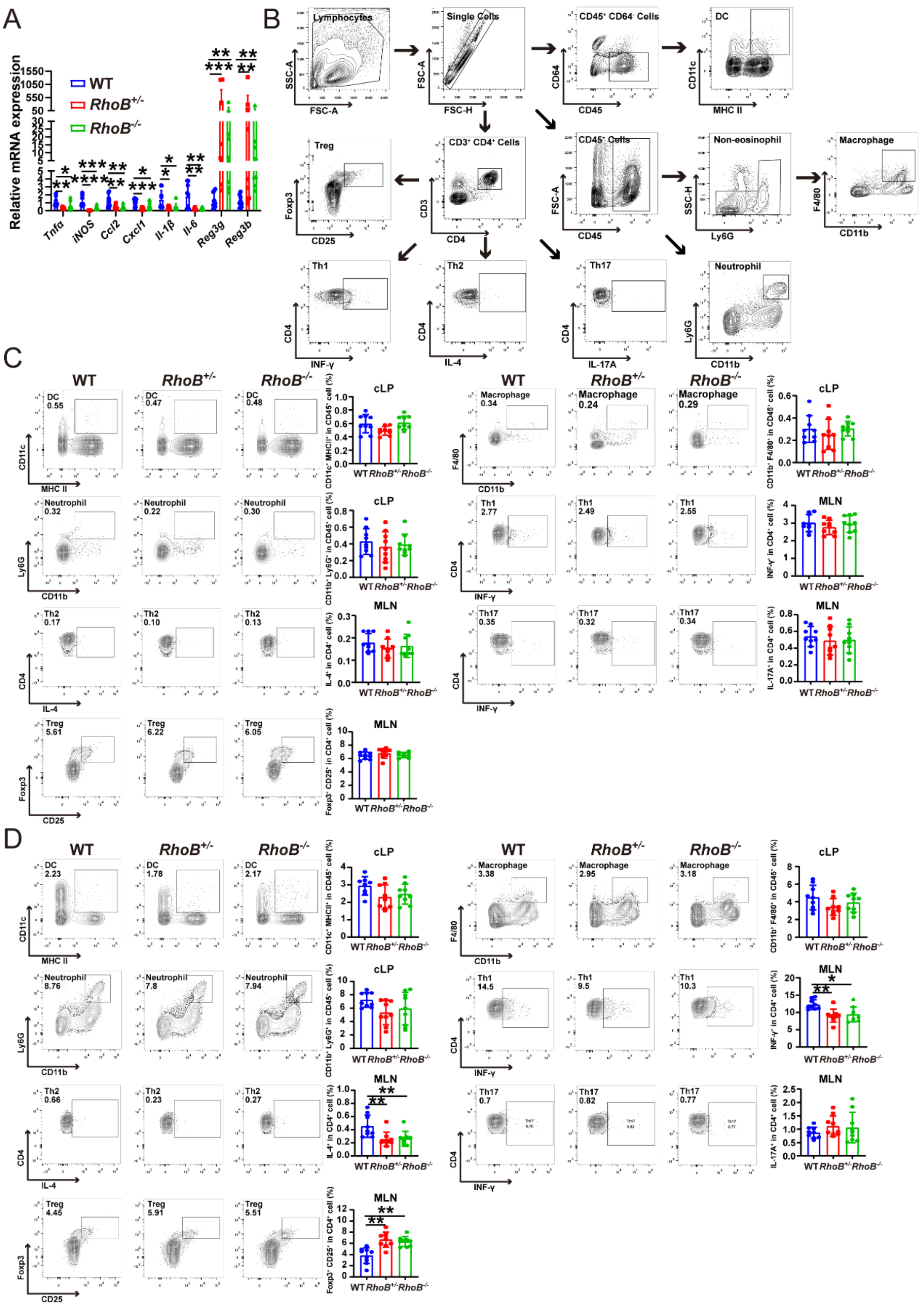

Figure S2. RhoB deficiency alleviates inflammatory responses to DSS treatment. (A) mRNA expression levels of anti-microbial peptides and proinflammatory cytokines in the colons of indicated mice measured by real-time PCR (n = 9 from 2 independent experiments). **(B)** Gating strategy for flow cytometry analysis of immune cells. **(C)** Representative flow cytometry analysis of the indicated cells (left) and percentage (right) of indicated cells in mesenteric lymph nodes (MLN) and colonic lamina propria

(cLP) from indicated naive mice (n = 9 from 2 independent experiments). **(D)** Representative flow cytometry analysis of the indicated cells (left) and percentage (right) of indicated cells in MLN and cLP from indicated mice after DSS administration (n = 9 from 2 independent experiments). Data are the mean ± SD. Statistical significance was determined by one-way ANOVA (A, C and D). *$p < 0.05$, **$p < 0.01$, ***$p < 0.001$.
