# Supplementary material for: RhoB affects colitis through modulating cell signaling and intestinal microbiome
Source: Microbiome. 2022 Sep 16;10:149. doi: 10.1186/s40168-022-01347-3 (PMC9482252; doi:10.1186/s40168-022-01347-3)
Supplement: Supplementary file 4 — Additional file 3: Figure S3. RhoB deficiency protects mice from DSS-induced chronic colitis. (A-F) Eight-week-old male mice were given 1% DSS in drinking water for seven days and followed by normal drinking water for an additional 7 days with 3 cycles of treatment (n = 9 from 2 independent experiments). (A) Schematic image illustrating chronic colitis design. (B) Body weight loss of the indicated mice and treatments. (C) Disease activity index of the indicated mice and treatments. (D) Measurement and quantification of colon length in the indicated mice. (E) Representative H&E staining analysis of histopathological changes and quantitation of histology score in colon from the indicated mice. (F) Alcian blue-Periodic acid Schiff (AB-PAS; indicating goblet cells) staining in colon from the indicated mice and treatments. Scale bar: 50 μm. (G) Measurement and quantification of colon length in naïve WT, RhoB+/-, and RhoB-/- mice (n = 9 from 3 independent experiments). (H) Representative H&E staining analysis of histopathological changes in colon from naïve WT, RhoB+/- and RhoB-/- mice. (I) Representative Lgr5, Occludin, ZO-1, and cleaved caspase-3 staining and quantitation in colon sections as indicated (n = 9 from 3 independent experiments). Scale bar: 50 μm. Data are the mean ± SD. Statistical significance was determined by two-way ANOVA (B and C) or Unpaired Student’s t-test (D-F) or one-way ANOVA (G and I). **p < 0.01, ***p < 0.001, ****p < 0.0001. NS, not significant. [file 40168_2022_1347_MOESM3_ESM.pdf]

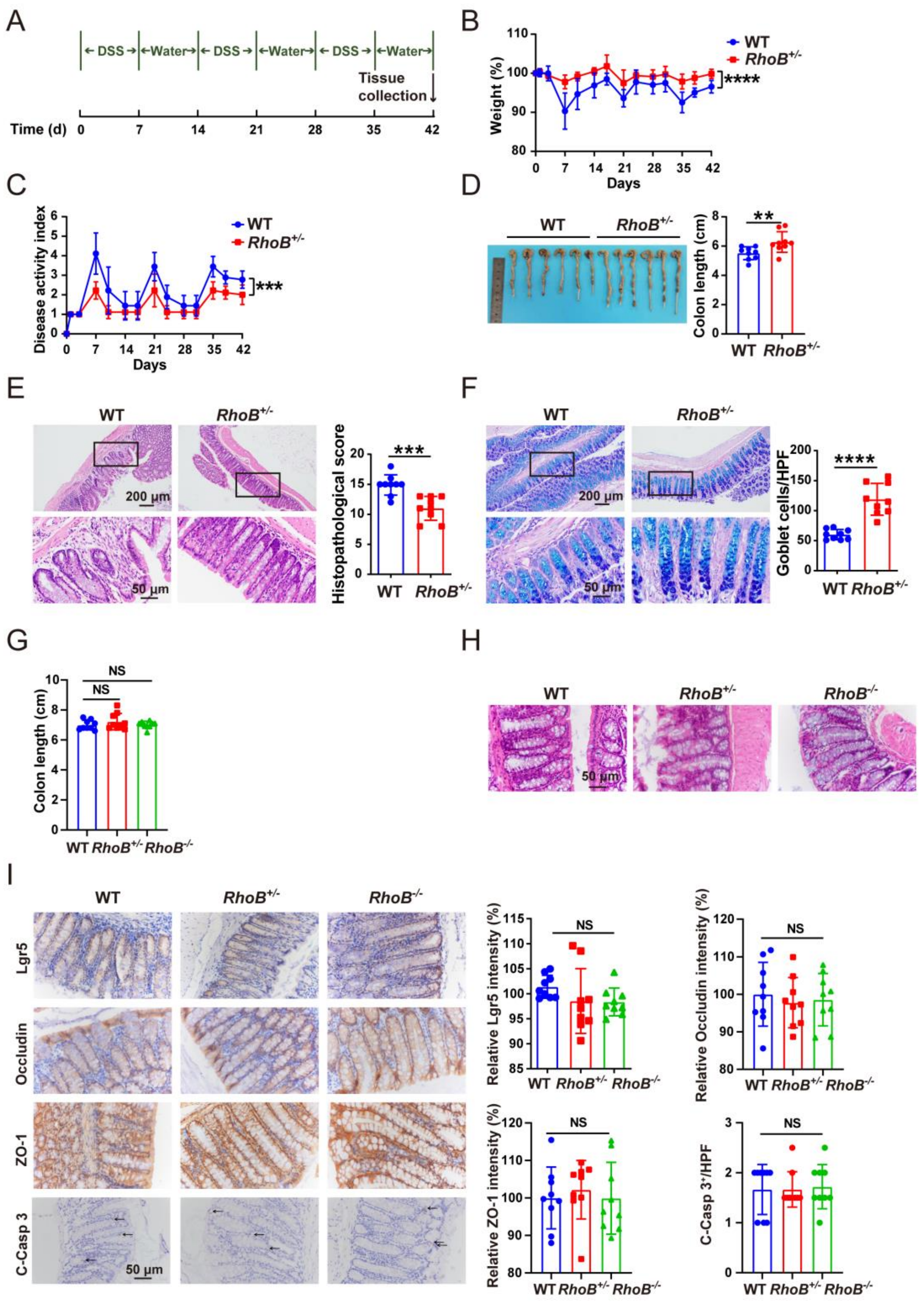

Figure S3. RhoB deficiency protects mice from DSS-induced chronic colitis. (A-F) Eight-week-old male mice were given 1% DSS in drinking water for seven days and followed by normal drinking water for an additional 7 days with 3 cycles of treatment (n = 9 from 2 independent experiments). **(A)** Schematic image illustrating chronic colitis design. **(B)** Body weight loss of the indicated mice and treatments. **(C)** Disease activity index of the indicated mice and treatments. **(D)** Measurement and quantification of colon length in the indicated mice. **(E)** Representative H&E staining analysis of histopathological

changes and quantitation of histology score in colon from the indicated mice. **(F)** Alcian blue-Periodic acid Schiff (AB-PAS; indicating goblet cells) staining in colon from the indicated mice and treatments. Scale bar: 50 μm. **(G)** Measurement and quantification of colon length in naïve WT, *RhoB*$^{+/-}$, and *RhoB*$^{-/-}$ mice (n = 9 from 3 independent experiments). **(H)** Representative H&E staining analysis of histopathological changes in colon from naïve WT, *RhoB*$^{+/-}$ and *RhoB*$^{-/-}$ mice. **(I)** Representative Lgr5, Occludin, ZO-1, and cleaved caspase-3 staining and quantitation in colon sections as indicated (n = 9 from 3 independent experiments). Scale bar: 50 μm. Data are the mean ± SD. Statistical significance was determined by two-way ANOVA (B and C) or Unpaired Student's t-test (D-F) or one-way ANOVA (G and I). $^{**}p < 0.01$, $^{***}p < 0.001$, $^{****}p < 0.0001$. NS, not significant.
