# Supplementary material for: RhoB affects colitis through modulating cell signaling and intestinal microbiome
Source: Microbiome. 2022 Sep 16;10:149. doi: 10.1186/s40168-022-01347-3 (PMC9482252; doi:10.1186/s40168-022-01347-3)
Supplement: Supplementary file 5 — Additional file 4: Figure S4. SB239063 and CHIR99021 could respectively restore increased expression of Ki67 and Muc2 in siRhoB transfected SW480 cells. (A) Western blotting analysis of RhoB knockdown efficiency in SW480 cells as indicated. β-Actin serves as a loading control. (B) Representative confocal images of Muc2 staining (red) and DAPI (blue) in SW480 cells and quantification. (C) Representative confocal images of Ki67 staining (green) and DAPI (blue) in SW480 cells and quantification. (D) Representative confocal images of Ki67 staining (green) and DAPI (blue) and quantification in SW480 cells as indicated. (E) Representative confocal images of Muc2 staining (red) and DAPI (blue) and quantification in SW480 cells as indicated. Scale bar: 100 μm (B-E). n = 3 from 3 independent experiments (B-E). Data are the mean ± SD. Unpaired Student’s t-test (B-C) or one-way ANOVA (D-E). **p < 0.01, ***p < 0.001, ****p < 0.0001. NS, not significant. [file 40168_2022_1347_MOESM4_ESM.pdf]

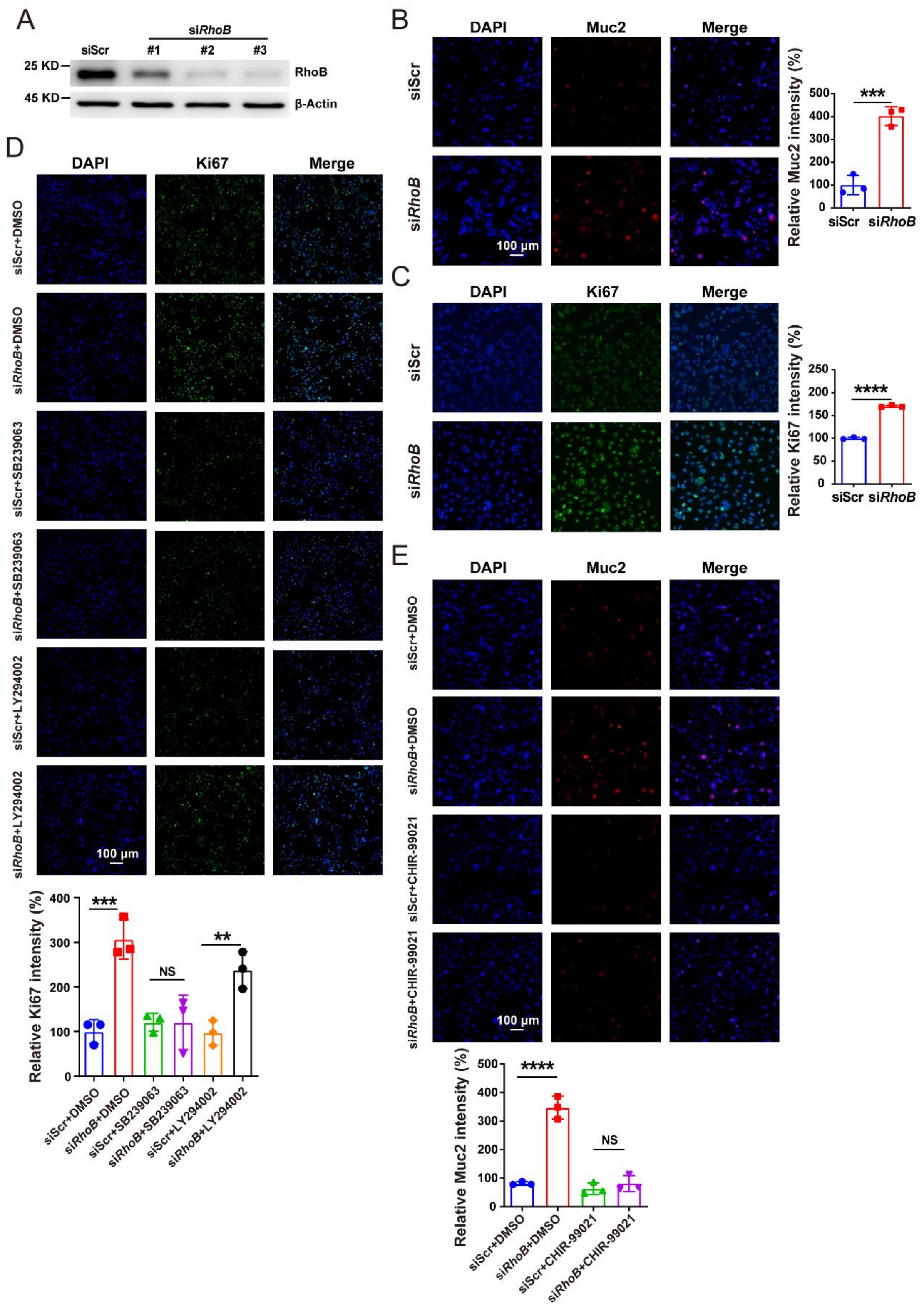

Figure S4. SB239063 and CHIR99021 could respectively restore increased expression of Ki67 and Muc2 in siRhoB transfected SW480 cells. (A) Western blotting analysis of *RhoB* knockdown efficiency in SW480 cells as indicated. β-Actin serves as a loading control. **(B)** Representative confocal images of Muc2 staining (red) and DAPI (blue) in SW480 cells and quantification. **(C)** Representative confocal images of Ki67 staining (green) and DAPI (blue) in SW480 cells and quantification.

(D) Representative confocal images of Ki67 staining (green) and DAPI (blue) and quantification in SW480 cells as indicated. **(E)** Representative confocal images of Muc2 staining (red) and DAPI (blue) and quantification in SW480 cells as indicated. Scale bar: 100 μm (B-E). n = 3 from 3 independent experiments (B-E). Data are the mean ± SD. Unpaired Student's t-test (B-C) or one-way ANOVA (D-E). $^{**}p < 0.01$, $^{***}p < 0.001$, $^{****}p < 0.0001$. NS, not significant.
