# Supplementary material for: RhoB affects colitis through modulating cell signaling and intestinal microbiome
Source: Microbiome. 2022 Sep 16;10:149. doi: 10.1186/s40168-022-01347-3 (PMC9482252; doi:10.1186/s40168-022-01347-3)
Supplement: Supplementary file 6 — Additional file 5: Figure S5. Altered microbiota in RhoB+/- and RhoB-/- mice. Stool samples from 6-8 week old WT, RhoB+/- or RhoB-/- mice were collected and analyzed by 16S rRNA gene sequencing (n = 5). (A) The amounts of bacterial DNA. (B) Analysis of the Shannon diversity index of microbiota. (C) PCoA results based on unweighted UniFrac distances. (D) Relative abundance of bacteria by taxon-based analyses. (E) Heatmap to visualize the relative abundances of the 30 most predominant bacterial genera. [file 40168_2022_1347_MOESM5_ESM.pdf]

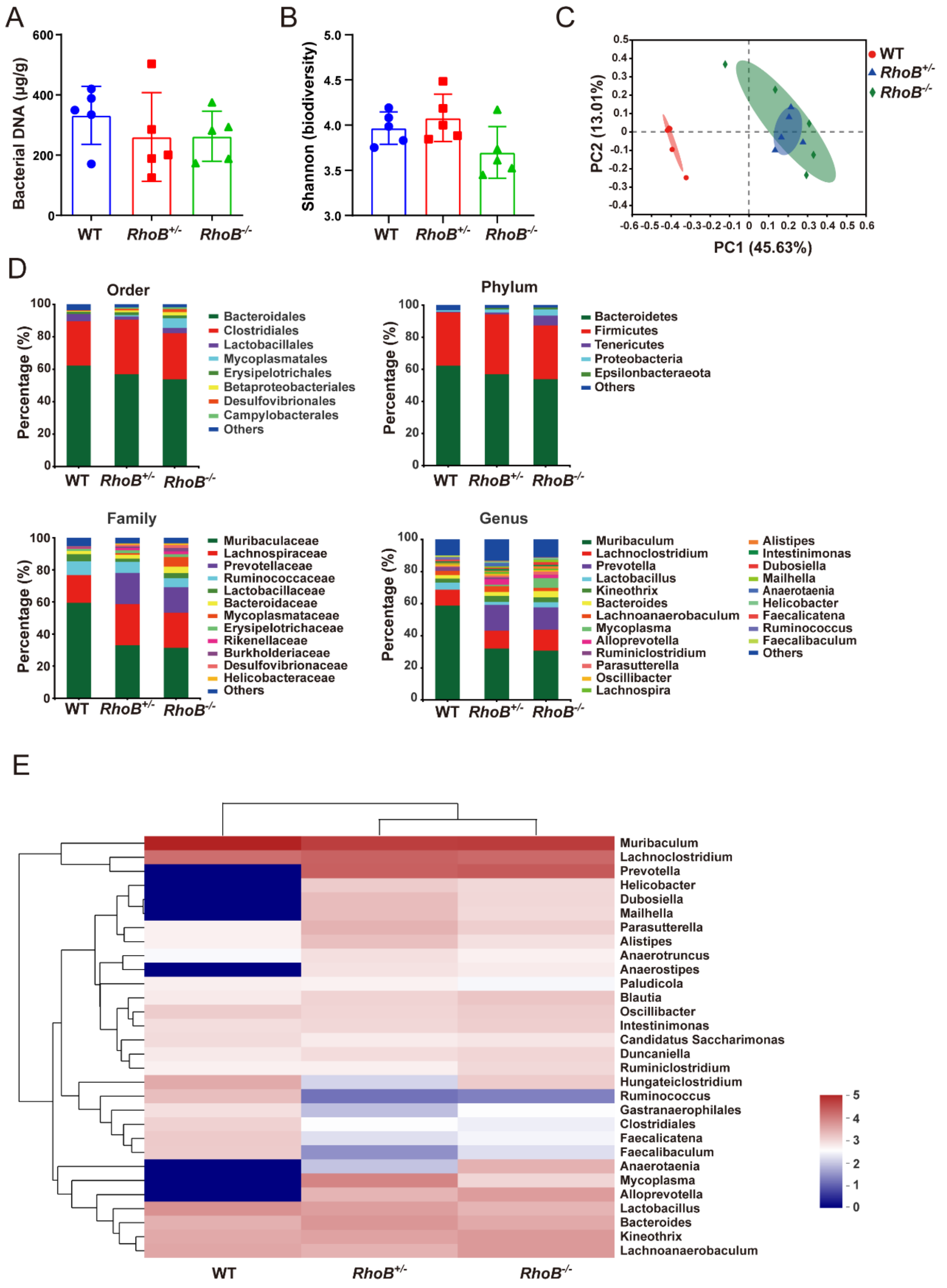

Figure S5. Altered microbiota in *RhoB*$^{+/-}$ and *RhoB*$^{-/-}$ mice. Stool samples from 6-8 week old WT, *RhoB*$^{+/-}$ or *RhoB*$^{-/-}$ mice were collected and analyzed by 16S rRNA gene sequencing (n = 5). **(A)** The amounts of bacterial DNA. **(B)** Analysis of the Shannon diversity index of microbiota. **(C)** PCoA results based on unweighted UniFrac distances. **(D)** Relative abundance of bacteria by taxon-based analyses. **(E)** Heatmap to visualize the relative abundances of the 30 most predominant bacterial genera.
