# Supplementary material for: RhoB affects colitis through modulating cell signaling and intestinal microbiome
Source: Microbiome. 2022 Sep 16;10:149. doi: 10.1186/s40168-022-01347-3 (PMC9482252; doi:10.1186/s40168-022-01347-3)
Supplement: Supplementary file 7 — Additional file 6: Figure S6. SCFA concentrations are positively correlated to the relative abundance of Prevotella and Alloprevotella. (A) Pearson’s correlation between the relative abundance of Prevotella and SCFAs in fecal from WT, RhoB+/-, and RhoB-/- mice (n = 15). (B) Pearson’s correlation between the relative abundance of Alloprevotella and SCFAs in fecal from WT, RhoB+/-, and RhoB-/- mice (n = 15). (C-F) Abx treatment eliminates the difference in the colitis phenotype of WT and RhoB-deficient mice (n = 5). (C) Scheme of Abx treatment protocol. (D) Disease activity index of WT and RhoB+/- mice. (E) Measurement and quantification of colon length in WT and RhoB+/- mice. (F) Representative H&E staining analysis of histopathological changes and quantitation of histology score in colons of WT and RhoB+/- mice. Boxed areas in colon sections are enlarged and shown at the bottom panel. Scale bar: 200 μm or 50 μm. Data are the mean ± SD. Significance is determined using linear regression (A-B) or two-way ANOVA (D) or unpaired Student’s t-test (E and F). NS, not significant. [file 40168_2022_1347_MOESM6_ESM.pdf]

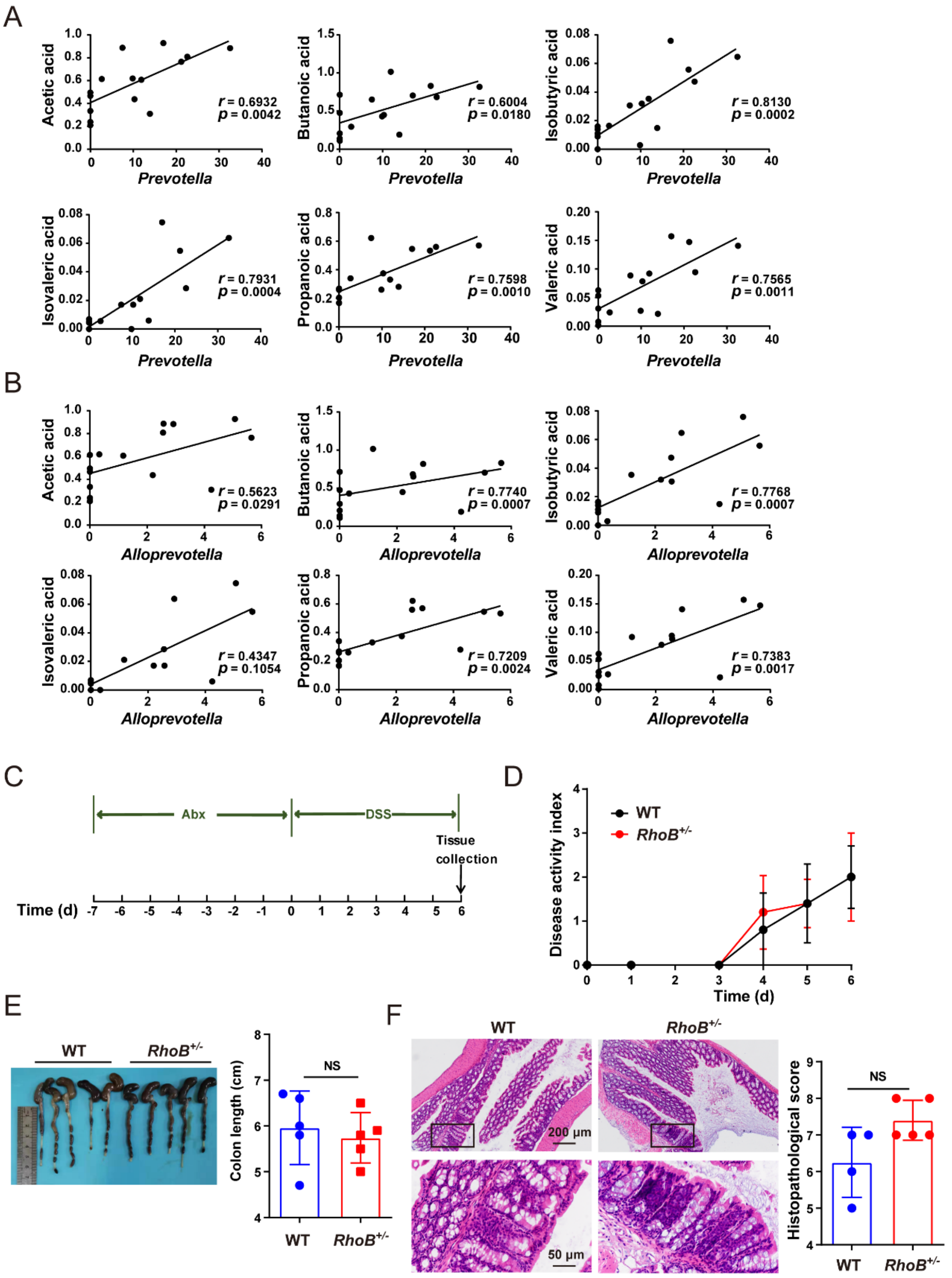

Figure S6. SCFA concentrations are positively correlated to the relative abundance of *Prevotella* and *Alloprevotella*. (A) Pearson's correlation between the relative abundance of *Prevotella* and SCFAs in fecal from WT, *RhoB*$^{+/-}$, and *RhoB*$^{-/-}$ mice (n = 15). **(B)** Pearson's correlation between the relative abundance of *Alloprevotella* and SCFAs in fecal from WT, *RhoB*$^{+/-}$, and *RhoB*$^{-/-}$ mice (n = 15). **(C-F)** Abx treatment eliminates the difference in the colitis phenotype of WT and RhoB-deficient mice (n = 5). **(C)** Scheme of Abx treatment protocol. **(D)** Disease activity index of WT and

RhoB$^{+/-}$ mice. **(E)** Measurement and quantification of colon length in WT and *RhoB*$^{+/-}$ mice. **(F)** Representative H&E staining analysis of histopathological changes and quantitation of histology score in colons of WT and *RhoB*$^{+/-}$ mice. Boxed areas in colon sections are enlarged and shown at the bottom panel. Scale bar: 200 μm or 50 μm. Data are the mean ± SD. Significance is determined using linear regression (A-B) or two-way ANOVA (D) or unpaired Student's t-test (E and F). NS, not significant.
