# Supplementary material for: RhoB affects colitis through modulating cell signaling and intestinal microbiome
Source: Microbiome. 2022 Sep 16;10:149. doi: 10.1186/s40168-022-01347-3 (PMC9482252; doi:10.1186/s40168-022-01347-3)
Supplement: Supplementary file 8 — Additional file 7: Figure S7. The microbiota composition in the feces from WT mice shifts toward that of RhoB-deficient mice after cohousing. Stool samples from WT and RhoB-deficient mice before and after cohousing were collected and analyzed by 16S rRNA gene sequencing (n = 4). (A) Analysis of the Shannon diversity index of microbiota in the indicated genotypes. (B) PCoA analysis of microbiota. (C) Relative abundance of fecal microbiota at the genus level. (D) LEfSe analysis of distinctive microbiota composition as indicated. [file 40168_2022_1347_MOESM7_ESM.pdf]

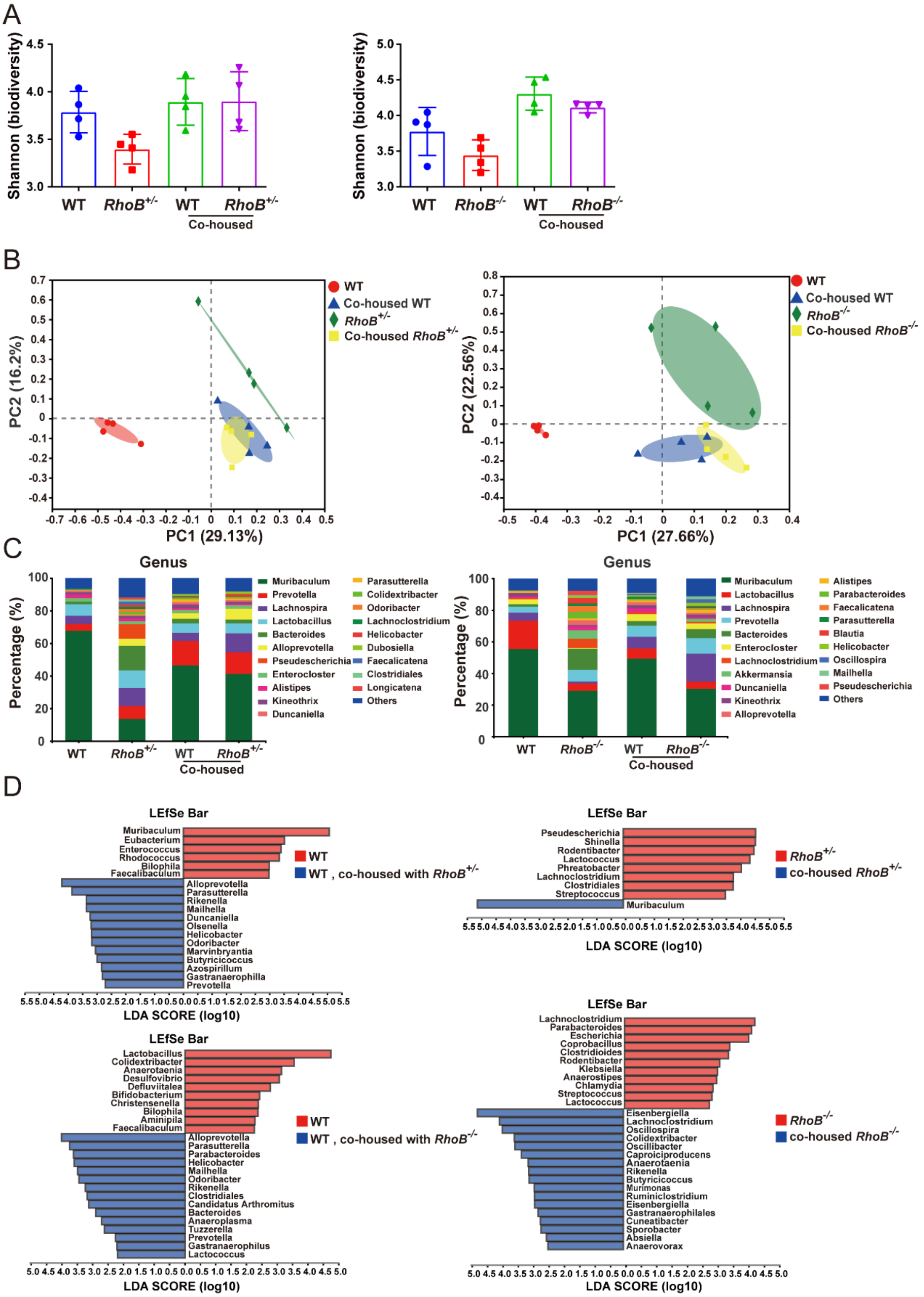

Figure S7. The microbiota composition in the feces from WT mice shifts toward that of RhoB-deficient mice after cohousing. Stool samples from WT and RhoB-deficient mice before and after cohousing were collected and analyzed by 16S rRNA gene sequencing (n = 4). **(A)** Analysis of the Shannon diversity index of microbiota in the indicated genotypes. **(B)** PCoA analysis of microbiota. **(C)** Relative abundance of fecal microbiota at the genus level. **(D)** LEfSe analysis of distinctive microbiota composition as indicated.
