# Supplementary material for: RhoB affects colitis through modulating cell signaling and intestinal microbiome
Source: Microbiome. 2022 Sep 16;10:149. doi: 10.1186/s40168-022-01347-3 (PMC9482252; doi:10.1186/s40168-022-01347-3)
Supplement: Supplementary file 9 — Additional file 8: Figure S8. Intestinal microbiota does not affect goblet cell numbers, Muc2 and Ki67 protein levels. (A-C) WT and RhoB-/- mice were cohoused for 4 weeks (n = 8 from 2 independent experiments). (A) Representative GPR41 and GPR43 staining and quantitation in colon sections as indicated. Scale bar: 50 μm. (B) Representative AB-PAS staining and quantification in colon sections of the indicated genotypes. (C) Representative Muc2 and Ki67 staining and quantitation in colon sections as indicated. (D-F) Autophagy induced by rapamycin does not reverse the colitis phenotype in RhoB-/- mice. Mice were treated with rapamycin for 7 days and then treated with 1.5% DSS (n = 4 or 5). (D) Disease activity index of the indicated genotypes. (E) Measurement and quantification of colon length in the indicated genotypes. (F) Histopathological changes and quantitation of histology score in colon of the indicated genotypes. Scale bar: 50 μm. Data are the mean ± SD. Unpaired Student’s t-test (A to C) or two-way ANOVA (D) or one-way ANOVA (E-F). *p < 0.05, **p < 0.01, ***p < 0.001. NS, not significant. [file 40168_2022_1347_MOESM8_ESM.pdf]

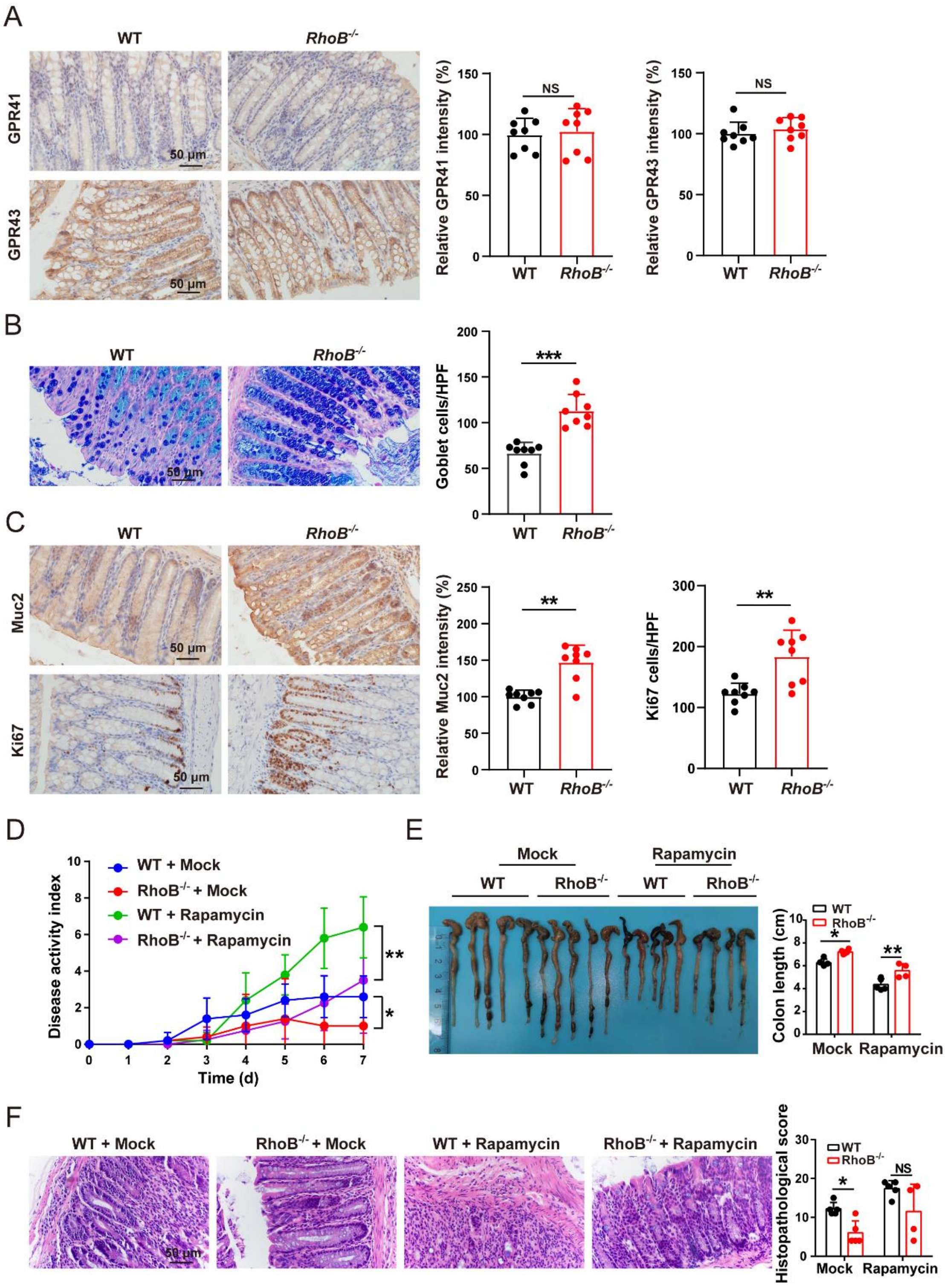

Figure S8. Intestinal microbiota does not affect goblet cell numbers, Muc2 and Ki67 protein levels. (A-C) WT and *RhoB*$^{-/-}$ mice were co-housed for 4 weeks (n = 8 from 2 independent experiments). **(A)** Representative GPR41 and GPR43 staining and quantitation in colon sections as indicated. Scale bar: 50 µm. **(B)** Representative AB-PAS staining and quantification in colon sections of the indicated genotypes. **(C)** Representative Muc2 and Ki67 staining and quantitation in colon sections as indicated. **(D-F)** Autophagy induced by rapamycin does not reverse the colitis phenotype in *RhoB*$^{-}$

/- mice. Mice were treated with rapamycin for 7 days and then treated with 1.5% DSS (n = 4 or 5). **(D)** Disease activity index of the indicated genotypes. **(E)** Measurement and quantification of colon length in the indicated genotypes. **(F)** Histopathological changes and quantitation of histology score in colon of the indicated genotypes. Scale bar: 50 μm. Data are the mean ± SD. Unpaired Student's t-test (A to C) or two-way ANOVA (D) or one-way ANOVA (E-F). *$p < 0.05$, **$p < 0.01$, ***$p < 0.001$. NS, not significant.
