# Supplementary material for: RhoB affects colitis through modulating cell signaling and intestinal microbiome
Source: Microbiome. 2022 Sep 16;10:149. doi: 10.1186/s40168-022-01347-3 (PMC9482252; doi:10.1186/s40168-022-01347-3)
Supplement: Supplementary file 10 — Additional file 9: Figure S9. The effect of RhoB on intestinal phenotype depends on its expression in epithelia. CD45.1 WT bone marrow were transferred into irradiated CD45.2 WT, RhoB+/- or RhoB-/- mice respectively (n = 6 from 2 independent experiments) (A) Scheme of bone marrow transplantation protocol. (B) Flow cytometry analysis of CD45.1 expression in peripheral blood cells after bone marrow transplantation. The percentage of CD45.1+cells in lymphocyte cells from the indicated genotypes. (C) Representative H&E staining analysis of histopathological changes in colon of the indicated genotypes. (D) Representative AB-PAS staining and quantification in colon sections of the indicated genotypes. (E) Representative confocal images and quantitation of Muc2 staining (green) and DAPI (blue) in colonic tissues of the indicated genotypes. (F) Ki67 staining in colon sections of the indicated genotypes. (G) Representative GPR41 and GPR43 staining and quantitation in colon sections as indicated. Scale bar: 50 μm or 10 μm. Data are the mean ± SD. One-way ANOVA (B-G). *p < 0.05, **p < 0.01, ***p < 0.001, ****p < 0.0001. [file 40168_2022_1347_MOESM9_ESM.pdf]

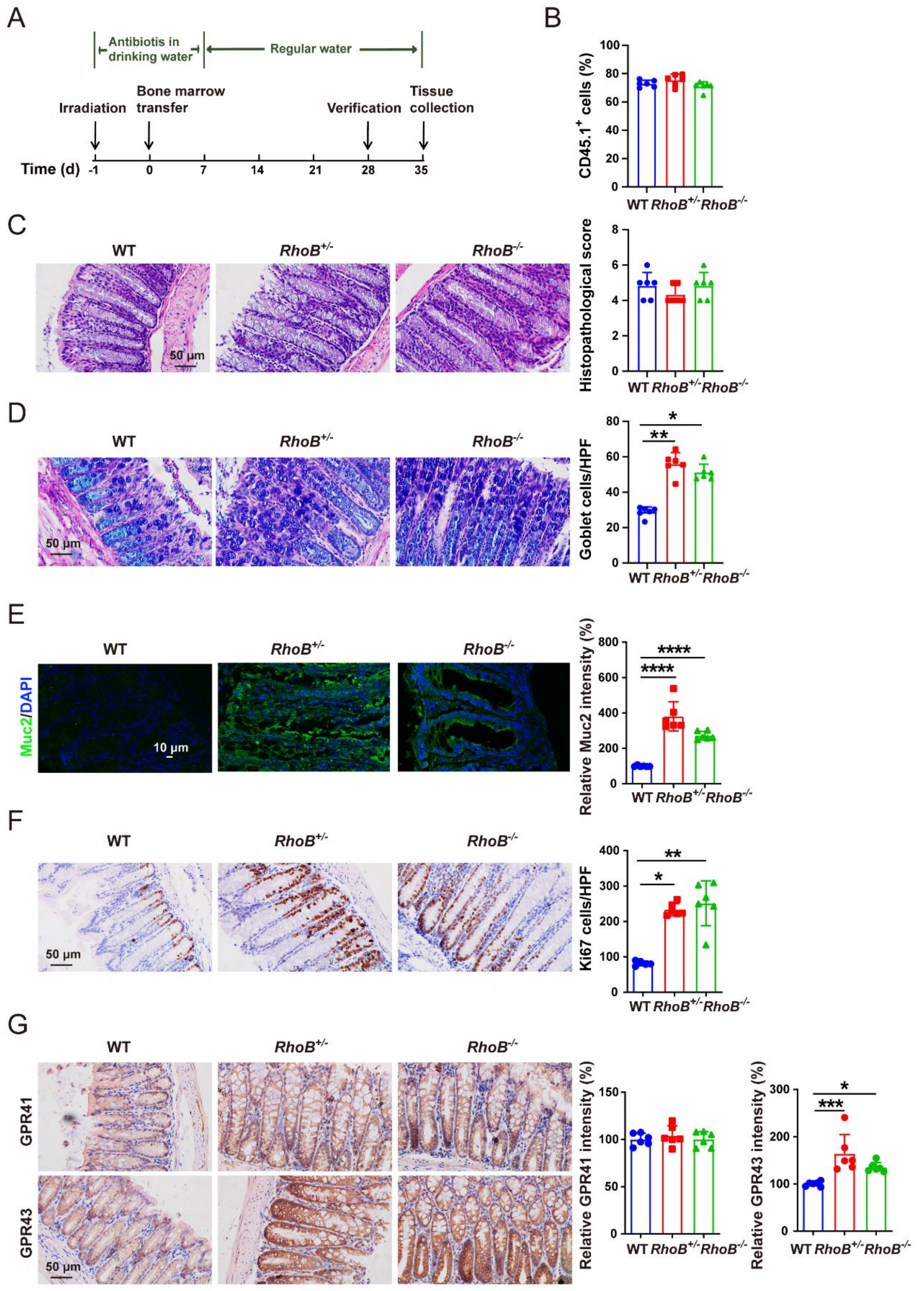

Figure S9. The effect of RhoB on intestinal phenotype depends on its expression in epithelia. CD45.1 WT bone marrow were transferred into irradiated CD45.2 WT, *RhoB*$^{+/-}$ or *RhoB*$^{-/-}$ mice respectively (n = 6 from 2 independent experiments) **(A)** Scheme of bone marrow transplantation protocol. **(B)** Flow cytometry analysis of CD45.1 expression in peripheral blood cells after bone marrow transplantation. The

percentage of CD45.1$^+$cells in lymphocyte cells from the indicated genotypes. **(C)** Representative H&E staining analysis of histopathological changes in colon of the indicated genotypes. **(D)** Representative AB-PAS staining and quantification in colon sections of the indicated genotypes. **(E)** Representative confocal images and quantitation of Muc2 staining (green) and DAPI (blue) in colonic tissues of the indicated genotypes. **(F)** Ki67 staining in colon sections of the indicated genotypes. **(G)** Representative GPR41 and GPR43 staining and quantitation in colon sections as indicated. Scale bar: 50 μm or 10 μm. Data are the mean ± SD. One-way ANOVA (B-G). *$p < 0.05$, **$p < 0.01$, ***$p < 0.001$, ****$p < 0.0001$.
