# Supplementary material for: RhoB affects colitis through modulating cell signaling and intestinal microbiome
Source: Microbiome. 2022 Sep 16;10:149. doi: 10.1186/s40168-022-01347-3 (PMC9482252; doi:10.1186/s40168-022-01347-3)
Supplement: Supplementary file 11 — Additional file 10: Figure S10. Autophagy does not contribute to microbiota alteration in RhoB-/- mice. Stool samples from WT and RhoB-/- mice before and after rapamycin treatment were collected and analyzed by 16S rRNA gene sequencing (n = 5). (A) Analysis of the Shannon diversity index of microbiota. (B) PCoA of microbiota. (C) Relative abundance of fecal microbiota at the genus level. (D) LEfSe analysis of distinctive microbiota composition as indicated. (E) Heatmap to visualize the relative abundances of the 30 most predominant bacterial genera. [file 40168_2022_1347_MOESM10_ESM.pdf]

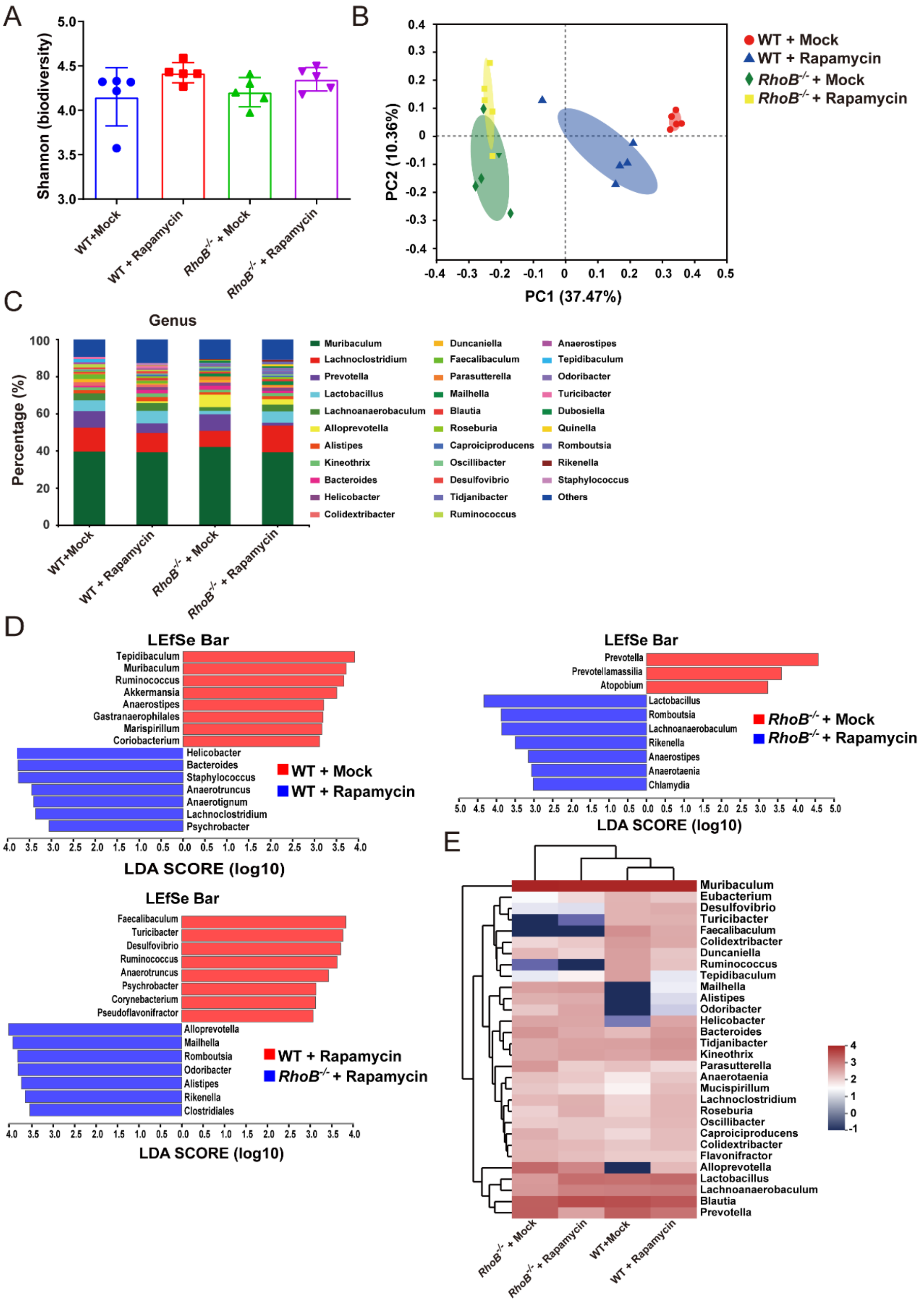

Figure S10. Autophagy does not contribute to microbiota alteration in *RhoB*$^{-/-}$ mice. Stool samples from WT and *RhoB*$^{-/-}$ mice before and after rapamycin treatment were collected and analyzed by 16S rRNA gene sequencing (n = 5). **(A)** Analysis of the Shannon diversity index of microbiota. **(B)** PCoA of microbiota. **(C)** Relative abundance of fecal microbiota at the genus level. **(D)** LEfSe analysis of distinctive microbiota composition as indicated. **(E)** Heatmap to visualize the relative abundances of the 30 most predominant bacterial genera.
