# Supplementary material for: RhoB affects colitis through modulating cell signaling and intestinal microbiome
Source: Microbiome. 2022 Sep 16;10:149. doi: 10.1186/s40168-022-01347-3 (PMC9482252; doi:10.1186/s40168-022-01347-3)
Supplement: Supplementary file 12 — Additional file 11: Figure S11. Autophagy does not affect Ki67, GPR41 and GPR43 protein levels. Mice were treated with rapamycin for 7 days and then were analyzed (n = 8 from 2 independent experiments). (A) Representative AB-PAS staining and quantification in colonic tissues of the indicated genotypes. (B) Representative Muc2 staining and quantitation in colon sections as indicated. (C) Representative confocal images of Muc2 staining in colonic tissues of the indicated genotypes. Quantification of the inner mucus layer thickness measured from the top of the villi to the periphery of the Muc2-positive region. Muc2: green; DAPI: blue. (D) Representative Ki67 staining and quantitation in colon sections as indicated. (E) Representative GPR41 staining and quantitation in colon sections as indicated. (F) Representative GPR43 staining and quantitation in colon sections as indicated. Scale bar: 10, 50 or 200 μm. Data are the mean ± SD. One-way ANOVA. ****p < 0.0001. NS, not significant. [file 40168_2022_1347_MOESM11_ESM.pdf]

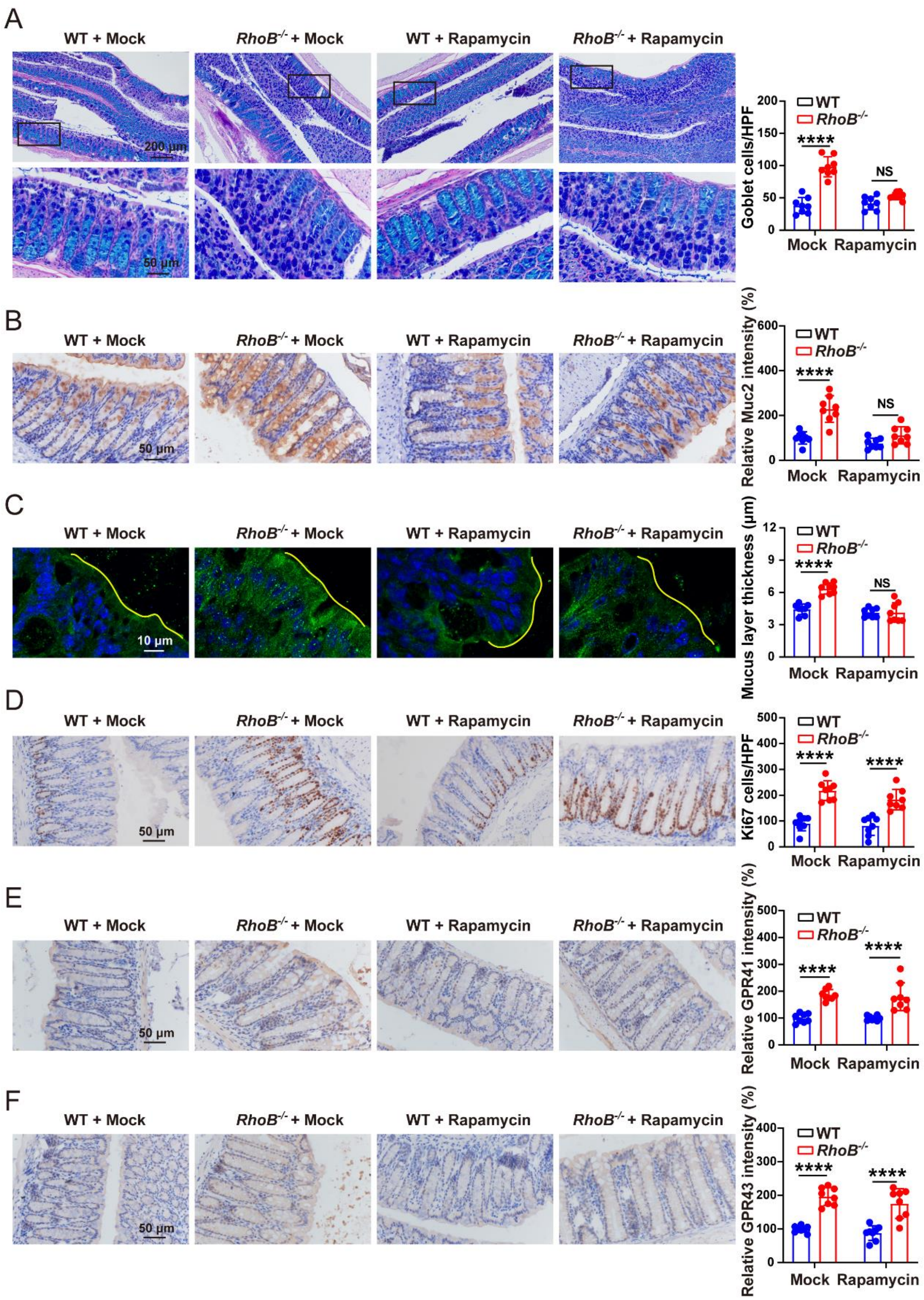

Figure S11. Autophagy does not affect Ki67, GPR41 and GPR43 protein levels. Mice were treated with rapamycin for 7 days and then were analyzed (n = 8 from 2 independent experiments). **(A)** Representative AB-PAS staining and quantification in colonic tissues of the indicated genotypes. **(B)** Representative Muc2 staining and quantitation in colon sections as indicated. **(C)** Representative confocal images of Muc2 staining in colonic tissues of the indicated genotypes. Quantification of the inner mucus layer thickness measured from the top of the villi to the periphery of the Muc2-positive

region. Muc2: green; DAPI: blue. **(D)** Representative Ki67 staining and quantitation in colon sections as indicated. **(E)** Representative GPR41 staining and quantitation in colon sections as indicated. **(F)** Representative GPR43 staining and quantitation in colon sections as indicated. Scale bar: 10, 50 or 200 µm. Data are the mean ± SD. One-way ANOVA. ****$p < 0.0001$. NS, not significant.
