# Supplementary material for: RhoB affects colitis through modulating cell signaling and intestinal microbiome
Source: Microbiome. 2022 Sep 16;10:149. doi: 10.1186/s40168-022-01347-3 (PMC9482252; doi:10.1186/s40168-022-01347-3)
Supplement: Supplementary file 13 — Additional file 12: Table S1. Characteristics of Patients with UC and Healthy Controls. [file 40168_2022_1347_MOESM12_ESM.pdf]

Table S1. Characteristics of Patients with UC and Healthy Controls

Participants	Age (year)	Sex	UC degree	CRP (mg/L)
1	26	male	mild	-
2	50	male	mild	-
3	20	female	mild	0.25
4	51	male	mild	-
5	68	female	mild	-
6	33	female	mild	-
7	19	female	mild	0.19
8	50	female	mild	0.5
9	30	male	mild	0.13
10	52	female	severe	-
11	39	male	severe	1.17
12	50	male	severe	1.71
13	65	male	severe	0.23
14	70	male	severe	-
15	23	female	severe	1.08
16	36	female	severe	2.36
17	50	female	severe	0.58
18	38	female	severe	3.46
19	47	female	severe	1.01
20	39	female	-	-
21	67	male	-	-
22	55	male	-	-
23	50	male	-	-
24	65	male	-	-
25	56	male	-	-
26	75	female	-	-
27	58	female	-	-
28	50	male	-	-
