# Supplementary material for: RhoB affects colitis through modulating cell signaling and intestinal microbiome
Source: Microbiome. 2022 Sep 16;10:149. doi: 10.1186/s40168-022-01347-3 (PMC9482252; doi:10.1186/s40168-022-01347-3)
Supplement: Supplementary file 14 — Additional file 13: Table S2. Primers used in this study. [file 40168_2022_1347_MOESM13_ESM.pdf]

Table S2. Primers used in this study

Primer	Sequences (5'-3')	Function
P1	GGACTGCAGCGGGCGACCAAT	Mouse genotype definition
P2	TCTGCCTCCGAAAATAAAATC	
P3	GGCTGCCTGCTCCACGAT	Mouse genotype definition
P4	GGCAGGGGCGCAGGGGCATAC	
TNFα-F	GATCGGTCCCCAAAGGGATG	For TNFα qRT-PCR
TNFα-R	TTTGCTACGACGTGGGCTAC	
IL-6-F	AGACAAAGCCAGAGTCCTTCAG	For IL-6 qRT-PCR
IL-6-R	AGACAAAGCCAGAGTCCTTCAG	
Cxcl1-F	TGGCTGGGATTCACCTCAAG	For Cxcl1 qRT-PCR
Cxcl1-R	CCGTTACTTGGGGACACCTT	
Ccl2-F	CACCAGCCAACTCTCACTGAA	For Ccl2 qRT-PCR
Ccl2-R	CATTCCTTCTTGGGGTCAGC	
iNOS-F	GTTCTCAGCCCAACAATACAAGA	For iNOS qRT-PCR
iNOS-R	GTGGACGGGTCGATGTCAC	
IL-1β-F	GGGCTGGACTGTTTCTAATGC	For IL-1β qRT-PCR
IL-1β-R	CTTGTGACCCTGAGCGACC	
Reg3b-F	AATGGAGGTGGATGGGAATG	For Reg3b qRT-PCR
Reg3b-R	CCACAGAAAGCACGGTCTAA	
Reg3g-F	CTTCCTGTCCTCCATGATCAAA	For Reg3g qRT-PCR
Reg3g-R	CCACCTCTGTTGGGTTCATAG	
β-Actin-F	CACTGTCGAGTCGCGTCCA	For β-Actin qRT-PCR
β-Actin-R	GACCCATTCCCACCATCACA	
N.C siRNA sense	GCGACGAUCUGCCUAAGAUdTdT	*RhoB* knockdown
N.C siRNA anti-sense	AUCUUAGGCAGAUCGUCGCdTdT	
siRNA-1 sense	GCUGAUCGUGUUCAGUAAGTT	*RhoB* knockdown
siRNA-1 anti-sense	CUUACUGAACACGAUCAGCTT	
siRNA-2 sense	CCGUCUUCGAGAACUAUGUTT	*RhoB* knockdown
siRNA-2 anti-sense	ACAUAGUUCUCGAAGACGGTT	

siRNA-3 sense	ACGUCAUUCUCAUGUGCUUTT	*RhoB* knockdown
siRNA-3 anti-sense	AAGCACAUGAGAAUGACGUTT	
A. rava-sense	TGAGGCATCTTGTTGCAACT	Quantitation of bacteria
*A. rav*a- anti-sense	GATGGTACACTCCAGAGGCG	
P. denticola-sense	CTTTACTGGGGTCCAGCCCGTCGAA	Quantitation of bacteria
P. denticola-anti-sense	CGCACTCTCATGGTTGAGCCACAAAAT	
16s rRNA-sense	GCCAGCAGCCGCGGTAA	Quantitation of bacteria
16s rRNA- anti-sense	AGGGTATCTAATCCT	
338F	ACTCCTACGGGAGGCAGCAG	Amplifying the V3-V4 hypervariable regions of the bacteria 16S rRNA gene
806R	GGACTACHVGGGTWTCTAAT	
Probes for *P. denticola*	GCCCAGTCGCCTGGTGGCTTCCC	Quantitation of bacteria
Probes for *A. rav*a	TGCTGCGGTTATTAACCGCAACA	
